# In Vivo Assembly of Nanoparticles Achieved through Synergy of Structure‐Based Protein Engineering and Synthetic DNA Generates Enhanced Adaptive Immunity

**DOI:** 10.1002/advs.201902802

**Published:** 2020-02-27

**Authors:** Ziyang Xu, Megan C. Wise, Neethu Chokkalingam, Susanne Walker, Edgar Tello‐Ruiz, Sarah T. C. Elliott, Alfredo Perales‐Puchalt, Peng Xiao, Xizhou Zhu, Ruth A. Pumroy, Paul D. Fisher, Katherine Schultheis, Eric Schade, Sergey Menis, Stacy Guzman, Hanne Andersen, Kate E. Broderick, Laurent M. Humeau, Kar Muthumani, Vera Moiseenkova‐Bell, William R. Schief, David B. Weiner, Daniel W. Kulp

**Affiliations:** ^1^ The Vaccine and Immunotherapy Center The Wistar Institute Philadelphia PA 19104 USA; ^2^ Department of Pharmacology Perelman School of Medicine University of Pennsylvania Philadelphia PA 19104 USA; ^3^ Inovio Pharmaceuticals Plymouth Meeting Philadelphia PA 19422 USA; ^4^ Department of Immunology and Microbiology The Scripps Research Institute La Jolla CA 92037 USA; ^5^ IAVI Neutralizing Antibody Center The Scripps Research Institute La Jolla CA 92037 USA; ^6^ Center for HIV/AIDS Vaccine Immunology and Immunogen Discovery The Scripps Research Institute La Jolla CA 92037 USA; ^7^ Bioqual Rockville MD 20852 USA; ^8^ Ragon Institute of MGH MIT and Harvard Cambridge MA 02139 USA; ^9^ Department of Microbiology Perelman School of Medicine University of Pennsylvania Philadelphia PA 19104 USA

**Keywords:** DNA vaccines, in vivo self‐assembly, infectious diseases, nanoparticle vaccines, protein engineering

## Abstract

Nanotechnologies are considered to be of growing importance to the vaccine field. Through decoration of immunogens on multivalent nanoparticles, designed nanovaccines can elicit improved humoral immunity. However, significant practical and monetary challenges in large‐scale production of nanovaccines have impeded their widespread clinical translation. Here, an alternative approach is illustrated integrating computational protein modeling and adaptive electroporation‐mediated synthetic DNA delivery, thus enabling direct in vivo production of nanovaccines. DNA‐launched nanoparticles are demonstrated displaying an HIV immunogen spontaneously self‐assembled in vivo. DNA‐launched nanovaccines induce stronger humoral responses than their monomeric counterparts in both mice and guinea pigs, and uniquely elicit CD8+ effector T‐cell immunity as compared to recombinant protein nanovaccines. Improvements in vaccine responses recapitulate when DNA‐launched nanovaccines with alternative scaffolds and decorated antigen are designed and evaluated. Finally, evaluation of functional immune responses induced by DLnanovaccines demonstrates that, in comparison to control mice or mice immunized with DNA‐encoded hemagglutinin monomer, mice immunized with a DNA‐launched hemagglutinin nanoparticle vaccine fully survive a lethal influenza challenge, and have substantially lower viral load, weight loss, and influenza‐induced lung pathology. Additional study of these next‐generation in vivo‐produced nanovaccines may offer advantages for immunization against multiple disease targets.

## Graphical abstract

Synthetic DNA cassettes, when combined with adaptive electroporation, are found to be able to launch in vivo assembly of supramolecular nanoparticle vaccines to induce superior adaptive immune responses and confer improved protection from viral challenges. It can now be envisioned that protein engineering can synergize with SynDNA technology for rapid de novo generation and evaluation of designer nanovaccines.

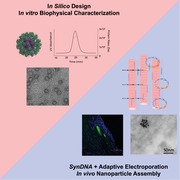

## 1 Introduction

Vaccination is an extremely important public health measure that has demonstrated prophylactic and therapeutic utility against many infectious diseases,^1^, ^2^, ^3^ and impacted some forms of cancer.^4^ In the past decade, advances in material engineering have allowed for the development and study of a new generation of nanoparticle vaccines.^5^, ^6^, ^7^ Hepatitis B and human papillomavirus (HPV) vaccines are such examples of self‐assembling virus‐like particles (VLPs) that have impacted millions of people.^8^, ^9^ Nanoparticles may come in several shapes and forms. Inorganic materials,^10^, ^11^ nontoxic phospholipids,^12^ VLPs, or self‐assembling protein nanoparticles (SAPN)^13^, ^14^, ^15^, ^16^ can scaffold and present antigens in repetitive multimeric manners to robustly stimulate immunity in animal models.^16^, ^17^, ^18^

However, some intrinsic production challenges have impeded broader translation of nanovaccines into the clinical space.^19^, ^20^ VLP vaccines are often produced at low yields in mammalian cell lines and are difficult to purify, requiring complex reassembly processes and additional post‐hoc characterization.^13^, ^21^, ^22^ Production of HPV VLPs, for example, requires three sequential purification steps of strong cation exchange chromatography, size‐exclusion chromatography (SEC), and hydroxyapatite chromatography.^23^ Large‐scale production of liposome‐based nanovaccines is challenging, as slight variations in the methods of production result in heterogeneity of the liposomes produced.^19^ Production of nanovaccines for a global market could, therefore, requires specialized pipelines that raise costs. In addition, regulatory approval of drugs for use in humans can be complex for the development of multicomponent nanomedicines.^24^ Technologies that would allow de novo nanoparticle assemblies in the hosts from materials that are inexpensive, simple, and stable, which bypass these complex biochemical processes and downstream purifications, may be of interest.

In this regard, computational protein engineering is an extremely powerful tool and has facilitated the design of novel biologics^25^ as well as specific potent nanovaccines.^15^, ^26^ One such example is the eOD‐GT8‐60mer, which is a priming immunogen engineered to activate precursors of HIV‐1 broadly neutralizing antibodies.^27^, ^28^, ^29^ When scaffolded with the C‐terminus of the lumazine synthase (LS) enzyme from *Aquifex aeolicus*, eOD‐GT8 can assemble into a 60 mer nanoparticle to induce stronger humoral immunity and higher frequencies of antigen‐specific IgG^+^ memory B cells.^27^ In terms of vaccine delivery, DNA vaccines have been studied for induction of humoral and cellular immunity.^30^, ^31^, ^32^ Additionally, delivery of optimized DNA plasmids encoding monomeric immunogens via adaptively controlled electroporation (EP)^33^ can result in 1000‐fold enhancement of in vivo expression and longer‐term in vivo production of the encoded antigens.^34^, ^35^, ^36^ The newer DNA platform is also a robust method of eliciting adaptive immune responses in humans, having demonstrated immune potency in the clinic against such diseases as Zika, Ebola, HIV, Middle East respiratory syndrome, and clinical efficacy against HPV‐driven cervical dysplasia.^4^, ^37^, ^38^, ^39^, ^40^

While simple multimerization domains, such as heptamer domain IMX313P, have been employed to improve DNA vaccine responses,^41^, ^42^ we explored and expanded upon this concept focusing on induction of both B‐ and T‐cell responses to large computationally designed nanoparticles (24, 60, and 180 mers) decorated with a variety of antigens. eOD‐GT8‐60mer is currently being clinically evaluated as a recombinant protein vaccine,^43^ and was examined as a prototype for DNA delivery. We discovered that the DNA‐Launched nanoparticle Lumazine Synthase decorated with an anti‐HIV‐1 immunogen eOD‐GT8 (eOD‐GT8‐60mer in the literature,^29^ herein referred as DLnano_LS_GT8) could assemble in vivo into nanoparticles. DLnano_LS_GT8 induced stronger humoral responses than corresponding DNA‐launched monomeric GT8 (DLmono_GT8) both in mice and guinea pigs, and also uniquely elicited CD8+ T‐cell immunity unlike the corresponding protein nanovaccines. We computationally designed alternative nanoparticle scaffolds and utilized different immunogens to evaluate this approach more broadly. Consistent improvements in the induction of adaptive immune responses were observed across multiple constructs, which was further shown to confer significant benefits in protecting mice from lethal influenza challenge. Synthetic DNA/electroporation (DNA/EP) technology can, therefore, be used to direct in vivo assembly of computationally designed nanovaccines, which elicit more potent functional immunological responses. This combination is likely important for rapid development of vaccines and immunotherapies.

## 2 Results

### 2.1 DNA‐Launched GT8 Nanoparticles Expressed and Assembled In vitro and In vivo

**Figure 1:**
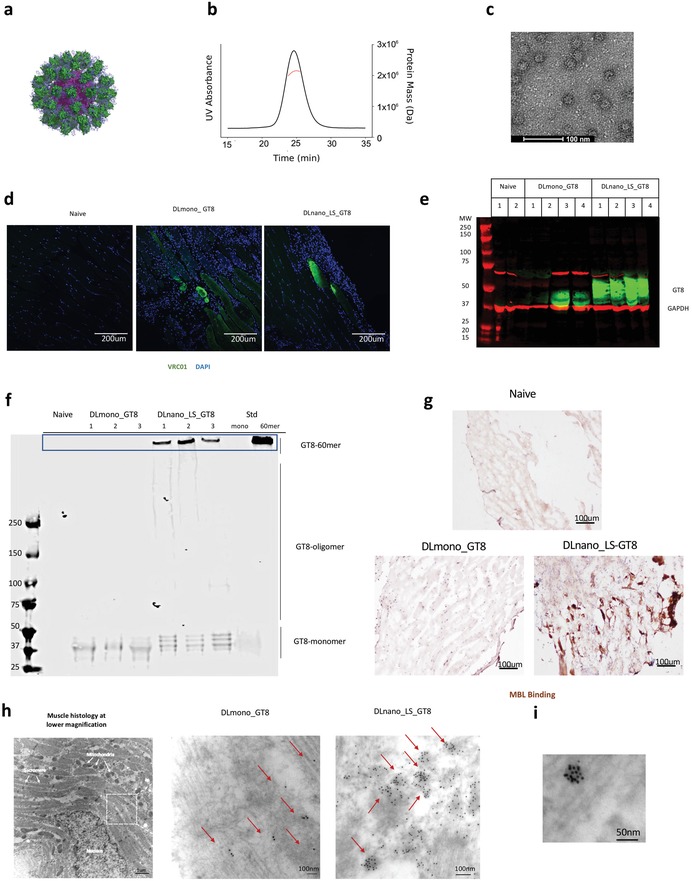
Expression and assembly of in vitro‐produced protein eOD‐GT8‐60mer and GT8‐monomer and in‐vivo produced DLnano_LS_GT8 and DLmono_GT8. a) Predicted structure of eOD‐GT8‐60mer. LS inner scaffold is shown in purple, decorated GT8 is shown in green, and N‐linked glycans are represented as blue sticks. b) SECMAL trace showing the calculated molecular weight of SEC purified eOD‐GT8‐60mer. c) Negative stain electron microscopy images of purified eOD‐GT8‐60mer. d) In vivo expression of DLmono_GT8 or DLnano_LS_GT8 in BALB/c mice 4 d.p.i., as probed by VRC01 and anti‐human Alexa Fluor 488. Nuclei staining with DAPI is shown in blue. e) Reducing SDS‐PAGE Western analysis to determine in vivo expression of DLmono_GT8 and DLnano_LS_GT8 4 d.p.i. in muscle homogenates with VRC01 (in green); GAPDH (in red) is used as the loading control. f) Pseudo‐native PAGE analysis comparing migration of in vivo‐produced DLmono_GT8 and DLnano_LS_GT8 to in vitro‐produced SEC‐purified recombinant GT8‐monomer (labeled as STD mono) and eOD‐GT8‐60mer (labeled as STD nano) protein standards. g) Murine MBL labeling of naïve mouse muscles or muscles transfected with DLmono_GT8 and DLnano_LS_GT8 7 d.p.i. h) Transmission electron microscopy (TEM) images of muscle sections from mice injected with DLmono_GT8 or DLnano_LS_GT8 7 d.p.i. that were immunolabeled with VRC01 and gold anti‐human IgG. Red arrows highlight VRC01 staining. i) TEM image of muscle section showing an example of high‐valency GT8 nanoparticle assembled in vivo. A total of 80 µg plasmid DNA dose of DLmono_GT8 or DLnano_LS_GT8 used in panels (d–i).

To determine whether DNA/EP could be used to launch structurally designed, SAPN in vivo, we encoded the transgene eOD‐GT8‐60mer in the pVAX1 vector and optimized the DNA cassette for in vivo nanoparticle expression (**Figure** **1**a). We first evaluated expression, secretion, and assembly of plasmid encoded GT8 constructs in vitro. We engineered the GT8 constructs to incorporate an optimized human IgE‐leader sequence^34^ and found the in vitro intracellular expression of this construct to be strongly enhanced as compared to GT8 constructs without any leader sequence (Figure S1a, Supporting Information). We therefore used the IgE constructs for subsequent experiments. In addition, reducing SDS‐PAGE analysis of transfection supernatants supported that both plasmid‐encoded GT8‐monomer and eOD‐GT8‐60mer could be secreted (Figure S1b, Supporting Information). Lectin‐purified protein eOD‐GT8‐60mer eluted as a homogenous fraction by SEC (Figure S1c, Supporting Information). The assembled protein was observed to be approximately 2 MDa as determined by protein conjugate analysis with size‐exclusion multiangle light scattering (SEC‐MALS) (Figure 1b). Negative stain electron microscopy (nsEM) also supported correct assembly of protein eOD‐GT8‐60mer with a diameter of around 32 nm (Figure 1c).

Next, we examined the in vivo expression of both DNA‐encoded GT8 monomer and nanoparticle constructs. Immunofluorescent staining of mouse muscles transfected with DNA/EP 4 days post‐injection (d.p.i.) with VRC01 (a human broadly neutralizing antibody with high‐affinity for GT8) showed that both DNA‐encoded GT8 constructs expressed in vivo (Figure 1d). Reducing SDS‐PAGE Western analyses of muscle homogenates 4 d.p.i. with VRC01 (in green) also confirmed in vivo expression of GT8 antigens, even though in vivo expression of DLnano_LS_GT8 was stronger and more consistent than DLmono_GT8 (Figure 1e). The assembly states of in vivo‐produced DLnano_LS_GT8 as compared to DLmono_GT8 in mouse muscle homogenates was examined with pseudo‐native PAGE. Well‐formed 60 mer GT8‐nanoparticles, as defined by the migration pattern of SEC‐purified recombinant protein eOD‐GT8‐60mer standard, was observed only in DLnano_LS_GT8‐treated but not in DLmono_GT8‐treated mice (Figure 1f). Bands that corresponded to monomeric and oligomeric GT8 band were also observed in DLnano_LS_GT8 muscle homogenates but were significantly less intense than the 60 mer band and may represent newly synthesized GT8‐subunits or partially assembled GT8‐oligomer transiting through cellular secretory networks.

Next, we used a mannose‐binding lectin (MBL) labeling experiment to assess for in vivo antigen multimerization and nanoparticle assembly. MBL is a protein that can form hexamer and preferentially bind to highly repeated glycan structures on a pathogen/antigen surface.^44^ A recent study by Tokatilian et al. demonstrated that only highly multimerized glycan structures (eOD‐GT8‐60mer but not eOD‐GT8‐monomer) could bind to MBL.^26^ In our study, we similarly showed using ELISA that while VRC01 could bind to both protein GT8‐60mer and GT8‐monomer, murine MBL could only bind to protein GT8‐60mer but not protein GT8‐monomer (Figure S1d,e, Supporting Information). Using this assay as multimerization readout, we demonstrated that in vivo‐produced DLnano_LS_GT8, but not DLmono_GT8, could bind to MBL (Figure S1f,g, Supporting Information). Further, we observed that DLnano_LS_GT8 could be strongly labeled by endogenous murine MBL via an immunohistochemistry experiment (Figure 1g).

As an additional way to assess in vivo nanoparticle formation, we employed a transmission electron microscopy‐based technique, where thin sections of transfected muscles were stained with VRC01 and gold‐conjugated anti‐human IgG. Clusters of gold‐labeled macromolecules suggestive of in vivo‐launched nanoparticles decorated with multiple copies of GT8 were only observed in mice injected with DLnano_LS_GT8 but not with DLmono_GT8 (Figure 1h; Figure S1h, Supporting Information). In DLnano_LS_GT8‐immunized mice, these clusters often had a valency greater than 10 (Figure 1i). We expected some reduction in labeling valency due to both steric hindrance in binding of VRC01 to individual GT8 subunits and limited solvent exposure on nanoparticle surfaces with thin sample sectioning. Quantitative measurements of the orders of clusters in different fields of interests demonstrated that partially formed (orders between 5 and 8) and well‐formed (orders no less than 9) nanoparticles were significantly more frequent in mice treated with DLnano_LS_GT8 than with DLmono_GT8, confirming in vivo assembly of these complex nanovaccines (Figure S1i, Supporting Information).

### 2.2 DLnano_LS_GT8 Elicited More Rapid Seroconversion and Higher Setpoint Antibody Titers than DLmono_GT8 and Similar Titers to Protein eOD‐GT8‐60mer

**Figure 2:**
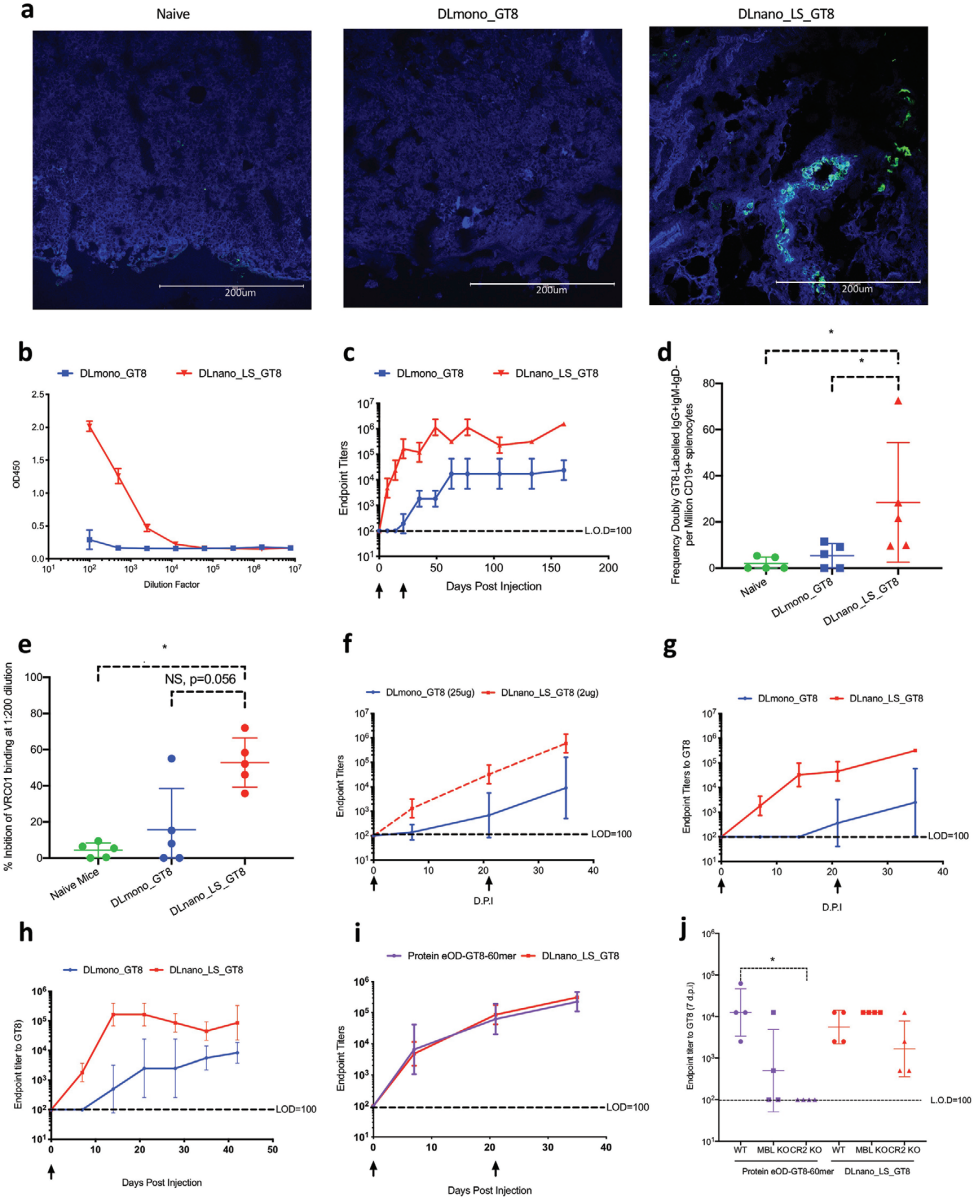
Characterization of in vivo trafficking of DLnano_LS_GT8 and humoral responses induced by DLnano_LS_GT8 versus DLmono_GT8. a) Trafficking of DLnano_LS_GT8 and DLmono_GT8 7 d.p.i. in the draining lymph nodes, as determined by VRC01 staining (green) and anti‐CD35‐BV421 staining (blue) for co‐localization analyses. b) ELISA binding against monomeric GT8 using serum from female BALB/c immunized with DLmono_GT8 or DLnano_LS_GT8 7 d.p.i. c) Endpoint titers to GT8 over time using serum from female BALB/c receiving two immunizations of DLmono_GT8 or DLnano_LS_GT8 3 weeks apart. d) Frequencies of CD19+IgM‐IgD‐IgG+ GT8‐specific B‐cells in the spleen of naïve female BALB/c mice or female BALB/c mice immunized with two doses of DLmono_GT8 or DLnano_LS_GT8 5 weeks after the second immunization. e) Percentage inhibition of VRC01‐GT8 binding by naïve mice sera or post‐immune sera from the DLmono_GT8 or DLnano_LS_GT8 vaccinated mice at 1:200 dilution. f) Comparison of GT8 endpoint titers for female BALB/c mice receiving two doses of DLmono_GT8 at 25 µg dose or DLnano_LS_GT8 at 2 µg dose. g) Comparison of GT8 endpoint titers for male BALB/c mice receiving two doses of DLmono_GT8 or DLnano_LS_GT8 at 25 µg dose. h) Comparison of endpoint titers in guinea pigs receiving single 50 µg intradermal immunization of DLmono_GT8 or DLnano_LS_GT8. i) Comparison of humoral responses induced by protein eOD‐GT8‐60mer adjuvanted by Sigma Adjuvant System or DLnano_LS_GT8 as assessed in C57BL/6 mice. j) Humoral responses in wildtype C57BL/6, MBL KO, or CR2 KO mice to protein eOD‐GT8‐60mer (purple) and DLnano_LS_GT8 vaccinations (red) 7 d.p.i. A total of 80 µg of plasmid DNA used in panel (a) and 25 µg plasmid DNA and 10 µg recombinant protein used elsewhere in the figure unless otherwise specified. Each group except in panel (j) includes five animals; each group in panel (j) includes four animals; each dot represents an animal; error bar represents standard deviation; arrow below the plot represents an immunization; two‐tailed Mann–Whitney rank test was used to compare groups; *p*‐values were adjusted for multiple comparison where appropriate; *p < 0.05.

Using immunofluorescence staining with VRC01 (green), we determined that DLnano_LS_GT8 trafficked more efficiently to the draining lymph node and colocalized with the CD35+ follicular dendritic cells (in blue) in contrast with the DLmono_GT8 7 d.p.i. (**Figure** **2**a). This observation is consistent with recent findings on trafficking of recombinant protein nanoparticle vaccines.^26^ To determine whether improved immunogen trafficking correlated with enhanced adaptive immunity, we followed humoral responses in immunized BALB/c mice. After 7 d.p.i., we found that DLnano_LS_GT8 induced more rapid GT8‐directed seroconversion than DLmono_GT8 (Figure 2b). Decoration of the GT8‐antigens on the LS nanoparticle core is essential for the observed early response as cotransfection of mice muscles with 1:1 ratio of DLmono_GT8 and DNA‐encoded LS core (DLnano_LS_core) did not lead to seroconversion at this timepoint (Figure S2a, Supporting Information). We next examined whether GT8 scaffolded with a simpler multimerization domain, IMX313P, would perform similarly. Heptameric DNA‐encoded GT8‐IMX313P (DL_GT8_IMX313P) led to limited seroconversion at 7 d.p.i., but the induced antibody titer was 6.9‐fold lower than that of DLnano_LS_GT8 (Figure S2b, Supporting Information). Antigen‐specific circulating IgMs can play a role in protection from challenge.^45^ Here, we measured induced IgM responses and found that DLnano_LS_GT8 induced stronger IgM responses than DLmono_GT8 with two immunizations (Figure S2c, Supporting Information). Further, the IgG titers were 1.3‐log and 1.8‐log higher for DLnano_LS_GT8 with single immunization (Figure S2d, Supporting Information) or two immunizations (Figure 2c), respectively. Consistent with this observation, we found the frequency of CD19+IgD‐IgM‐IgG+ GT8 antigen‐specific B cells in the spleens of mice immunized with DLnano_LS_GT8 to be 5.3‐fold higher relative to mice immunized with DLmono_GT8 (Figure 2d), even though relatively few CD19+IgD‐IgM‐IgG+GT8‐24mer+GT8‐tetramer+ B cells have been recovered per million splenocytes analyzed (Figure S2e, Supporting Information). DLnano_LS_GT8 retained folding and presentation of a key conformational epitope in vivo, as elicited murine antibodies could outcompete VRC01 binding to GT8 in competition of ELISA (Figure S2f, Supporting Information, and Figure 2e). A striking dose‐sparing effect was observed for DLnano_LS_GT8. While humoral responses were remarkably attenuated for DLmono_GT8 at 2 and 10 µg doses (Figure S2g, Supporting Information), DLnano_LS_GT8 given at 2, 10, or 25 µg doses all induced similar levels of antibody responses (Figure S2h, Supporting Information). Importantly, differences in antibody responses induced by DLnano_LS_GT8 and DLmono_GT8 were probably not solely due to increased antigen expression for DLnano_LS_GT8 (Figure 1e), as DLnano_LS_GT8 still outperformed DLmono_GT8 at less than one‐tenth of the monomer dose (Figure 2f).

The ability of DLnano_LS_GT8 to improve humoral responses was observed in other animal models. Strikingly, two immunizations in C57BL/6 mice of DLmono_GT8 failed to induce seroconversion, while DLnano_LS_GT8 induced strong humoral responses (Figure S2i, Supporting Information). In genetically diverse CD1 mice, we also observed more rapid seroconversion and more robust responses for DLnano_LS_GT8 (Figure S2j, Supporting Information). Additionally, we observed DLnano_LS_GT8 significantly improved humoral responses in both female (Figure 2c) and male (Figure 2g) BALB/c mice relative to DLmono_GT8. Finally, in guinea pigs, a single 50 µg intradermal (ID) vaccination of DLnano_LS_GT8 remarkably induced seroconversion 7 d.p.i. and 1.2‐log higher antibody titers than DLmono_GT8 over time (Figure 2h). We proceeded with studies of ID vaccination in guinea pigs as ID delivery has additional advantages of simplicity, improved tolerability, and being dose sparing.^38^, ^40^

We next compared the antibody responses induced by protein eOD‐GT8‐60mer and DLnano_LS_GT8. Protein eOD‐GT8‐60mer was subcutaneously administered in mice to be consistent with prior studies involving administration of this immunogen to mice;^27^, ^28^ further, a relative high protein dose of 10 µg was used in this study as compared to prior study for protein versus DNA comparison.^26^ We observed that two sequential immunizations of protein eOD‐GT8‐60mer co‐formulated with Sigma Adjuvant System or DLnano_LS_GT8 in C57BL/6 mice induced similar humoral responses (Figure 2i). It has been recently reported that uptake and trafficking of protein‐based nanoparticles are dependent on the MBL complement pathway.^26^, ^46^ We explored whether DNA‐launched nanoparticles depended on a similar mechanism. Similar to previous reports,^26^ humoral responses elicited by protein‐based GT8 nanoparticles in transgenic MBL and CR2 knockout mice were attenuated as compared to the wildtype C57BL/6 mice 7 d.p.i. (Figure 2j). Strikingly, similar humoral responses were induced in the MBL or CR2 knockout mice as compared to the wildtype C57BL/6 mice by DLnano_LS_GT8 (Figure 2j), highlighting DLnano immunogens may act independently of MBL‐complement pathway, potentially through redundant mechanisms of antigen presentation.

### 2.3 DLnano_LS_GT8 Elicited Superior Cellular Responses than DLmono_GT8 and Uniquely Induced CD8+ T‐Cell Responses Relative to Protein eOD‐GT8‐60mer

**Figure 3:**
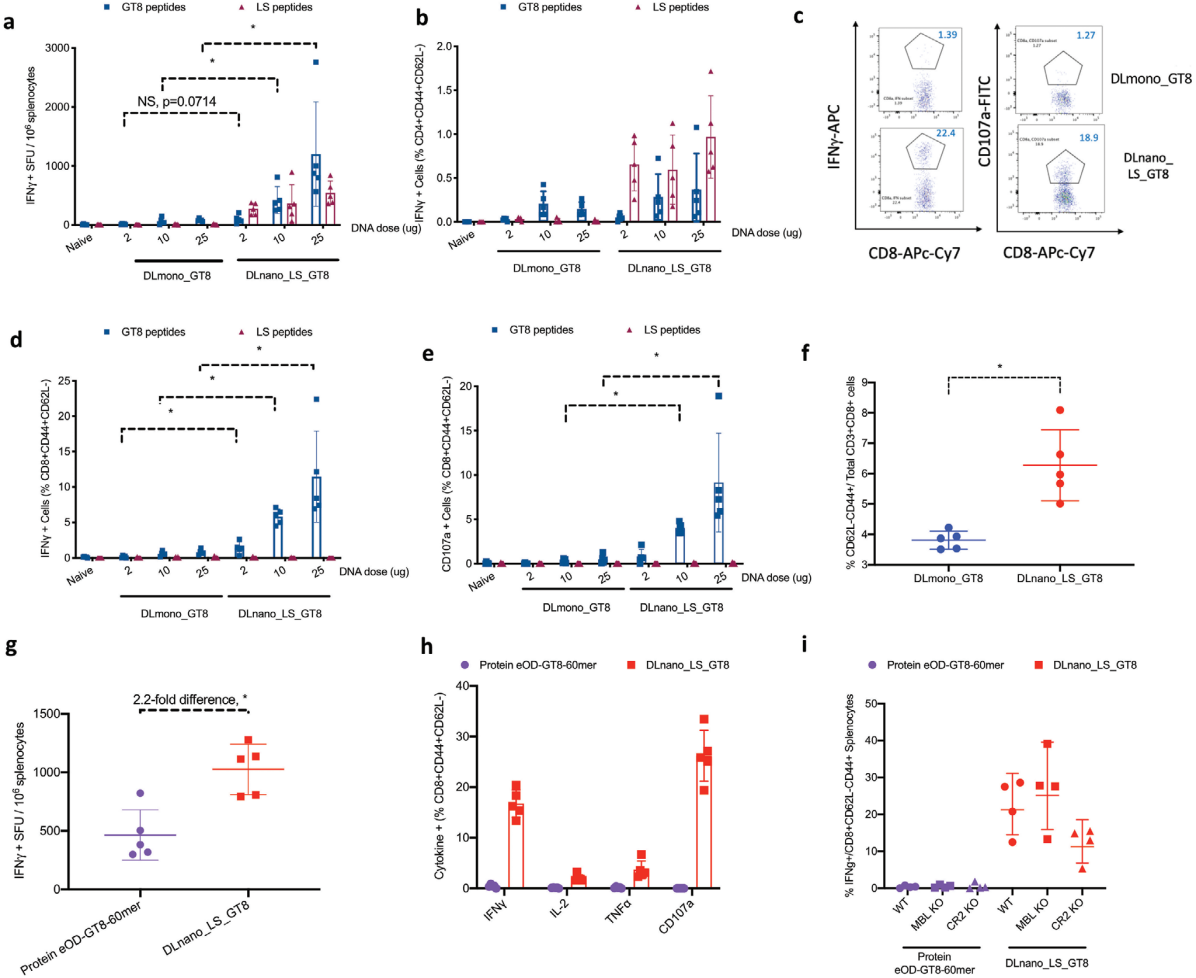
Characterization of cellular responses induced by DLnano_LS_GT8 versus DLmono_GT8 in BALB/c mice and by protein eOD‐GT8‐60mer and DLnano_LS_GT8 in C57BL/6 mice. a) ELIspot responses to the LS peptides and GT8 peptides in BALB/c mice immunized with two doses of DLmono_GT8 or DLnano_LS_GT8 at specified doses. b) Effector memory CD4+ T‐cell responses (CD3+CD4+CD44+CD62L‐) in immunized BALB/c mice as in panel (a). c–e) Effector memory CD8+ T‐cell responses (CD3+CD8+CD44+CD62L‐) in immunized BALB/c mice in terms of IFNγ expression in panel (d) and CD107a expression in panel (e). f) Comparison for the frequencies of CD8+ effector memory T‐cell responses induced by DLmono_GT8 or DLnano_LS_GT8 immunizations in BALB/c mice. g) T‐cell responses as determined by IFN‐γ ELISpot assays for protein eOD‐GT8‐60mer and DLnano_LS_GT8 immunized C57BL/6 mice. h) CD4+ effector memory T‐cell responses for protein eOD‐GT8‐60mer and DLnano_LS_GT8 immunized C57BL/6 mice as determined by ICS. i) Comparisons of CD8+ T‐cell responses induced by protein eOD‐GT8‐60mer (purple) versus DLnano_LS_GT8 vaccinations (red) in in wildtype C57BL/6, MBL KO or CR2 KO mice. A total of 25 µg plasmid DNA and 10 µg recombinant protein used in the figure unless otherwise specified. Each group except in panel (i) includes five mice; each group in panel (i) includes four animals; each dot represents a mouse; error bar represents standard deviation; two‐tailed Mann–Whitney rank test was used to compare groups; *p*‐values were adjusted for multiple comparison where appropriate; *p < 0.05.

We next examined the induction of antigen‐specific cellular responses by DNA nanovaccines. DLnano_LS_GT8 elicited significantly stronger antigen (GT8)‐specific cellular responses than DLmono_GT8 in BALB/c mice as determined by IFNγ‐ELIspot assays (**Figure** **3**a). Intracellular cytokine staining (ICS) revealed that the scaffolding LS domain drove predominantly CD4+ responses, since a higher proportion of effector memory CD3+CD4+CD44+CD62L‐ T‐cells produced IFNγ, TNFα, and IL‐2 when stimulated by the LS peptides than by GT8 peptides (Figure 3b; Figure S3a,b, Supporting Information). In contrast, we found that effector memory CD3+CD8+CD44+CD62L‐T cells induced by DLnano_LS_GT8 were more reactive to the GT8 domain than to the LS domain. DLnano_LS_GT8 induced more antigen‐specific effector memory CD8+ T‐cells that expressed activation cytokines IFNγ and exhibited effector phenotypes (CD107a+) than DLmono_GT8 in BALB/c mice (Figure 3c–e).

In C57BL/6 mice, we also found that DLnano_LS_GT8 elicited strong T‐cell responses to the full immunogen. Both CD4+ and CD8+ responses were predominant to the LS domain, possibly due to the lack of CD8+ T‐cell epitope in the GT8 domain for this inbred strain (Figure S3c–e, Supporting Information). To determine the ability of DLnano_LS_GT8 to elicit T‐cell responses to the antigenic domain in a model with more diverse HLA haplotypes, we used the outbred CD1 mice and found that DLnano_LS_GT8 induced stronger CD4+ and CD8+ effector memory T‐cell responses to the GT8 domain than DLmono_GT8 (Figure S3f–h, Supporting Information). In all mice strains studied, we observed DLnano_LS_GT8 elicited significantly higher frequencies of effector memory CD8+ T‐cells than could DLmono_GT8 (Figure 3f; Figure S3i, Supporting Information). Additionally, DLnano_LS_GT8 was observed to induce stronger CD8+ T‐cell responses to the GT8 domain in both female (Figure 3d) and male (Figure S3j, Supporting Information) BALB/c mice.

In comparison to protein eOD‐GT8‐60mer, we observed two immunizations of DLnano_LS_GT8‐induced 2.2‐fold higher T‐cell responses by IFNγ‐ELIspot assay (Figure 3g). In addition, ICS revealed that while both protein and DNA‐encoded GT8‐nanoparticles induced CD4+ responses (Figure S3k, Supporting Information), only DNA‐launched but not protein‐based nanoparticles elicited potent CD8+ T‐cell responses (Figure 3h; Figure S3l, Supporting Information). Recombinant protein nanoparticle failed to induce CD8+ T‐cell responses in both WT and transgenic MBL and CR2 knockout mice; whereas DLnano_LS_GT8 induced robust CD8+ T‐cell responses in these strains (Figure 3i), confirming our prior observations that DLnanovaccines may act independently of the MBL‐complement pathway.

### 2.4 Designed DNA‐Launched GT8‐Nanoparticles with Alternative Scaffolds Analogously Induced Improved Adaptive Immune Responses

**Figure 4:**
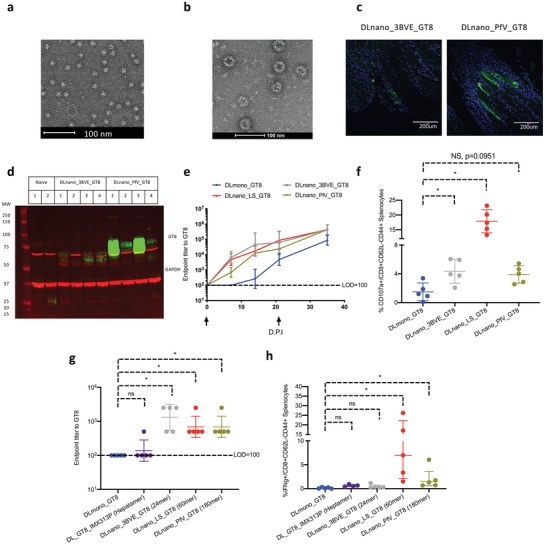
Design and evaluation of new DLnano GT8‐vaccines with alternative scaffolds. a) nsEM image of SEC‐purified fraction of in vitro‐produced 3BVE‐GT8 nanoparticles. b) nsEM image of SEC‐purified fraction of in vitro produced PfV‐GT8 nanoparticles. c) In vivo expression of DLnano_3BVE_GT8 and DLnano_PfV_GT8 in transfected mouse muscles as determined by immunofluorescence; VRC01 labeling is shown in green and nuclei labeling is shown in blue. d) Reducing SDS‐PAGE Western analysis to determine in vivo expression of DLnano_3BVE_GT8 and DLnano_PfV_GT8 4 d.p.i. in muscle homogenates with VRC01 (in green); GAPDH (in red) is used as the loading control. e) Humoral responses in BALB/c mice immunized with two 25 µg doses of DLmono_GT8, DLnano_3BVE_GT8, DLnano_LS_GT8, and DLnano_PfV‐GT8. f) CD8+ effector memory CD107a+ T‐cell responses to GT8 domain in BALB/c mice immunized with DLmono_GT8, DLnano_3BVE_GT8, DLnano_LS_GT8, and DLnano_PfV‐GT8 as in panel (e). g) Humoral responses in BALB/c mice immunized with 2 µg doses of DLmono_GT8, DL_GT8_IMX313P, DLnano_3BVE_GT8, DLnano_LS_GT8, and DLnano_PfV‐GT8 7 d.p.i. h) CD8+ effector memory CD107a+ T‐cell responses to GT8 domain in BALB/c mice immunized twice with 2 µg DLmono_GT8, DL_GT8_IMX313P, DLnano_3BVE_GT8, DLnano_LS_GT8, and DLnano_PfV‐GT8 3 weeks apart. A total of 80 µg of plasmid DNA used in panels (c and d); 25 µg plasmid DNA used elsewhere in panels (e and f); 2 µg plasmid DNA used in panels (g and h). Each group contains five mice; each dot represents a mouse; error bar represents standard deviation; arrow below the plot represents an immunization; two‐tailed Mann–Whitney rank test was used to compare groups; *p*‐values were adjusted for multiple comparison where appropriate; *p < 0.05.

To ensure that the observed phenomena were not limited to LS scaffolded nanoparticles, we computationally designed additional GT8 nanoparticles. Using the crystal structures of ferritin from *Helicobacter pylori* (3BVE, a 24 mer), and PfV viral cage from *Pyrococcus furiosus* (2e0z, a 180‐mer), we modeled GT8 at various geometries relative to the particle surface and designed appropriate flexible linkers. 3BVE‐GT8 homogeneously assembled into spherical nanoparticles by nsEM (Figure S4a, Supporting Information, and **Figure** **4**a). For PfV_GT8, we observed mixed species, but the predominant peak at 9.14 mL retention time, which accounted for approximately 60% of overall intensity, corresponded to torus‐shaped nanoparticle by nsEM (Figure S4b, Supporting Information, and Figure 4b). To demonstrate decoration of the designed nanoparticles with GT8, recombinantly produced protein 3BVE_GT8, eOD‐GT8‐60mer and PfV_GT8 were all tested and observed to bind to VRC01 (Figure S4c, Supporting Information). Immunofluorescence demonstrated that both DLnano_3BVE_GT8 and DLnano_PfV_GT8 expressed in vivo 4 d.p.i. (Figure 4c), even though in vivo expression of DLnano_PfV_GT8 was found to be stronger on average than DLnano_3BVE_GT8 by SDS‐PAGE analysis (Figure 4d). Functionally, BALB/c mice immunized with DLnano_3BVE_GT8, DLnano_LS_GT8 and DLnano_PfV_GT8 all rapidly seroconverted 7 d.p.i. and mounted stronger antibody responses over the 5 week period than mice immunized with DLmono_GT8 (Figure 4e). In addition, BALB/c mice immunized with two doses of DLnano_3BVE_GT8, DLnano_LS_GT8, and DLnano_PfV_GT8 all developed stronger CD8+ effector memory T‐cell responses to the antigenic GT8 domain than those immunized with DLmono_GT8 by IFNγ ELIspot and ICS assays (Figure 4f; Figure S4d,e, Supporting Information).

Valency of nanoparticles was found to be relevant to the dose‐sparing phenomenon observed (Figure 2f). At low DNA dose of 2 µg, we found that 24, 60, and 180‐meric DNA‐launched GT8 nanoparticle vaccines but not heptameric DL_GT8_IMX313P were capable of inducing seroconversion in BALB/c mice at 7 d.p.i. (Figure 4g). In terms of cellular immunity at this dose, we found that only 60‐ and 180‐meric but not hepta‐ and 24‐meric DNA‐launched GT8 nanovaccines were capable of inducing improvement in CD8+ T‐cell immunity relative to DLmono_GT8 (Figure 4h). Overall, we observed that the aforementioned nanoparticle domains can be designed to display antigens such as GT8 to elicit rapid and strong adaptive immune responses.

### 2.5 Designed DNA‐Launched Hemagglutinin Nanovaccine Induced Improved Functional Antibody Responses and Stronger CD8+ T‐Cell Immunity

**Figure 5:**
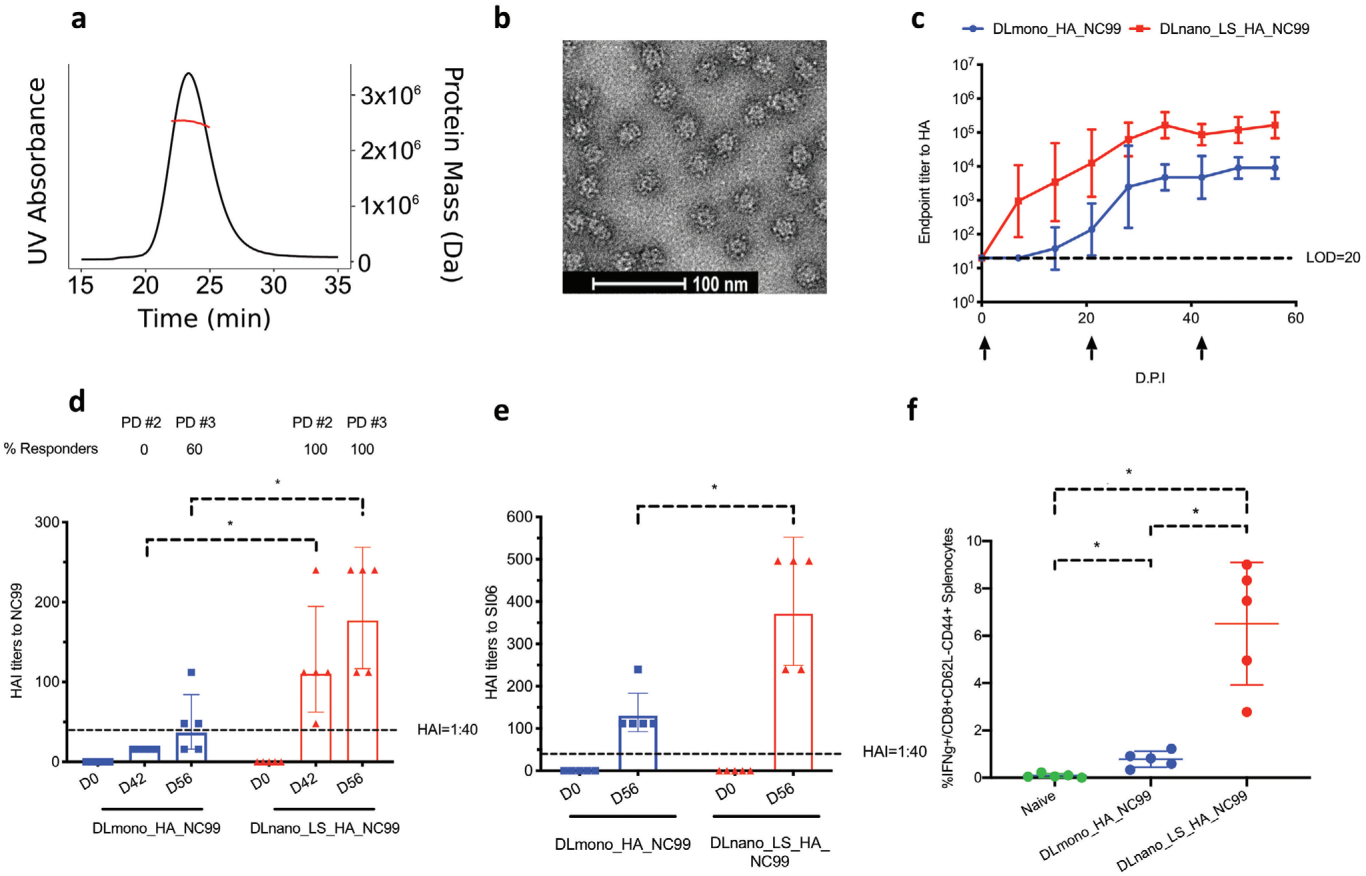
Design and evaluation of new DLnano influenza hemagglutinin vaccine. a) SECMAL trace of lectin and SEC purified LS_HA_NC99. b) nsEM image of SEC‐purified fraction of in vitro‐produced protein LS_HA_NC99 nanoparticles. c) Humoral responses in BALB/c mice that received DLnano_LS_HA_NC99 or DLmono_HA_NC99 at 1 µg dose. d) Autologous HAI titers against the H1 NC99 strain at D0, D42 (post‐dose #2) and D56 (post‐dose #3) for mice treated with 1 µg DLmono_HA_NC99 or DLnano_LS_HA_NC99. e) Heterologous HAI titers against the H1 SI06 strain at 56 d.p.i. for mice treated with 1 µg DLmono_HA_NC99 or DLnano_LS_HA_NC99. f) CD8+ effector memory IFNγ+ T‐cell responses to NC99 HA domain in naïve BALB/c mice or mice immunized with two doses of 10 µg DLmono_HA_NC99 or DLnano_LS_HA_NC99. Each group contains five mice; each dot represents a mouse; error bar represents standard deviation; arrow below the plot represents an immunization; two‐tailed Mann–Whitney rank test was used to compare groups; *p*‐values were adjusted for multiple comparison where appropriate; *p < 0.05.

To determine whether these findings could be applied to an immunogen relevant to a different infectious disease, we computationally designed a LS nanoparticle to display the receptor binding domain of the head of influenza hemagglutinin (LS_HA_NC99) based on the H1N1 strain A/New Caledonia/20/1999 and confirmed its assembly into homogenous 60‐mer by both SEC, SEC‐MAL, and nsEM (Figure S5a, Supporting Information, **Figure** **5**a,b). A dose‐sparing phenomenon was observed for DLnano_LS_HA_NC99, as at a remarkably low plasmid vaccine dose of 1 µg, DLnano_LS_HA_NC99 induced significantly stronger humoral responses in BALB/c mice than DLmono_HA_NC99 (Figure 5c). Hemagglutinin inhibition titers (HAI) against the autologous NC99 strain were found to be higher than 1:40 (which correlated with 50% reduction in the risk of infections in humans^47^) in 100% of mice immunized with two doses of DLnano_LS_GT8 and 0% in mice immunized with two doses of DLmono_HA_NC99 (Figure 5d). At the final timepoint (56 d.p.i.) after three immunizations, both the DLmono_HA_NC99 and DLnano_LS_HA_NC99 groups developed binding and HAI antibodies to the heterologous H1N1 influenza A/Solomon Island/3/06 strain (Figure S5b, Supporting Information, and Figure 5e), and both the binding and HAI titers were still significantly higher for the DLnano_LS_HA_NC99 group. HAI of a more distant H1 strain, A/California/07/2009, was not detected in either group.

Additionally, in terms of elicited cellular responses, two immunizations of DLnano_LS_HA_NC99 induced 8.4‐fold higher effector memory CD8+ T‐cell responses than DLmono_HA_NC99 at 10 µg dose in terms of CD107a and IFNγ expression, similar to our prior findings (Figure 5f; Figure S5c,d, Supporting Information).

Finally, we examined whether homogenous in vitro assembly of the designed DLnanovaccine was a prerequisite to its enhanced potency. To this end, we studied the in vivo properties of a poorly folded nanoparticle. We used an alternative LS scaffolded influenza construct, DNA‐encoded LS_HA_CA09, based on the A/California/07/2009 strain that did not pass our biophysical filters, as in vitro expression of the construct showed 3+ peaks with the two largest peaks consisting of aggregates or smaller unassembled protein by SEC (Figure S5e, Supporting Information). We found DNA‐encoded LS_HA_CA09 could not induce the characteristic early seroconversion in BALB/c mice (Figure S5f, Supporting Information). Even when the immunized mice were followed over time, the antibody responses induced by DNA‐encoded LS_HA_CA09 still lagged behind those by DLnano_LS_HA_NC99, highlighting downstream success of DLnanovaccine predicated upon preliminary computational design and biophysical characterization.

### 2.6 DNA‐Launched Hemagglutinin Nanovaccine Conferred Improved Protection to Lethal Pandemic Influenza H1 A/California/07/09 Challenge in Mice

**Figure 6:**
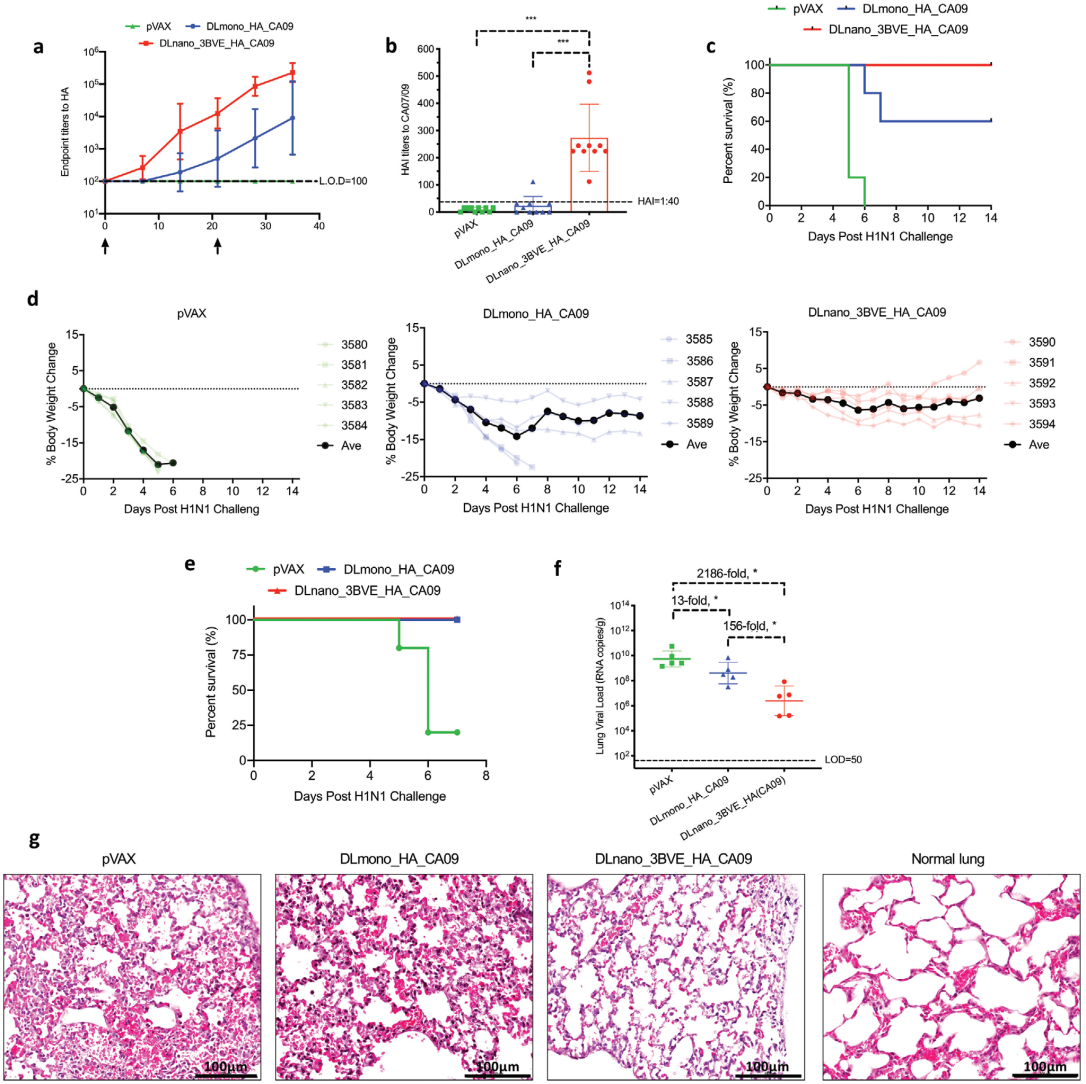
Functional evaluations of DLmono_HA_CA09 versus DLnano_3BVE_HA_CA09 in H1 A/California/07/09 lethal challenge model. a) Binding endpoint titers to HA (CA09) over time in BALB/c mice immunized with two 1 µg doses of pVAX, DLmono_HA_CA09, or DLnano_3BVE_HA_CA09 3 weeks apart. b) HAI titers to the autologous A/California/07/09 strain in BALB/c mice immunized with 1 µg pVAX, DLmono_HA_CA09, or DLnano_3BVE_HA_CA09 5 weeks from their first vaccination. c) Percentages of vaccinated mice surviving the lethal 10LD_50_ H1/A/California/07/09 challenge over 2 week period. d) Weight changes in mice immunized with pVAX, DLmono_HA_CA09, or DLnano_3BVE_HA_CA09 over 2 week period following 10LD_50_ H1/A/California/07/09 challenge. e) Percentages of vaccinated mice surviving the lethal 10LD_50_ H1/A/California/07/09 challenge over 7 day period in a separate study. f) Lung viral load in challenged mice at 7 days post‐challenge or at the time of euthanasia as determined by RT‐qPCR. g) H&E stain for lung histopathology in mice 7 days after viral challenge or at the time of euthanasia, normal lung histology is shown for comparison; scale bar represents 100 µm. Each group contained 10 mice in panels (a and b); each group contained five in the remaining panels; each dot represents a mouse; error bar represents standard deviation; arrow below the plot represents an immunization; two‐tailed Mann–Whitney rank test used to compare groups; *p*‐values were adjusted for multiple comparison where appropriate; *p < 0.05.

To further evaluate the induction of functional immune responses by DLnanovaccines, we utilized a lethal influenza challenge model in mice. We constructed a ferritin‐scaffolded receptor‐binding domain of hemagglutinin from H1/California/07/09 strain, DLnano_3BVE_HA_CA09, that was leader sequence, codon, and mRNA‐optimized as compared to a previously reported construct.^48^ We first confirmed its in vitro assembly into nanoparticles by SEC and nsEM (Figure S6a,b, Supporting Information). We then immunized three groups of mice twice with minimal doses (1 µg) of DNA encoding either DLmono_HA_CA09, DLnano_3BVE_HA_CA09, or control backbone pVAX vector 3 weeks apart. We observed improved induction of binding antibody responses in mice immunized with DLnano_3BVE_HA_CA09 than those with DLmono_HA_CA09 (**Figure** **6**a). Five weeks after the first immunization, we observed significant eightfold improvement in HAI titers in mice immunized with DLnano_3BVE_HA_CA09 than those with DLmono_HA_CA09 (Figure 6b). We then set up two lethal influenza challenge experiments in these three groups of mice, 5 weeks after the final immunization. Each mouse was intranasally inoculated with 10LD_50_ homologous H1/California/07/09 virus and was followed up for 2 weeks for weight loss. Any mouse losing more than 20% of baseline body weight would have met the humane endpoint for euthanasia. In this experiment, we observed only mice immunized with DLnano_3BVE_HA_CA09 fully survived the lethal challenge (Figure 6c), whereas 40% (2/5) of mice immunized with DLmono_HA_CA09 or 100% (5/5) of mice immunized with control pVAX backbone succumbed to infections. Additionally, among mice that survived the challenge, substantially lower weight loss was observed in mice immunized with DLnano_3BVE_HA_CA09 than DLmono_HA_CA09 (Figure 6d).

In a separate set of experiments, we followed these three groups of immunized mice 7 days post H1/CA09 challenge to determine lung viral load and pathology. It was observed, in this challenge study, that within the first 7 days, 80% (4/5) of mice immunized with control pVAX vector succumbed to infection, but mice immunized with either DLmono_HA_CA09 and DLnano_3BVE_HA_CA09 survived the first 7 days (Figure 6e), even though mice immunized with DLmono_HA_CA09 still lost substantially more weight than those immunized with DLnano_3BVE_HA_CA09 (Figure S6c, Supporting Information). Additionally, we observed significant reduction in viral load of mice immunized with DLnano_3BVE_HA_CA09 as compared to mice immunized with pVAX (2186‐fold reduction) or with DLmono_HA_CA09 (156‐fold reduction) (Figure 6f). Finally, H&E staining of lung specimens at 7 days after the challenge or at the time of euthanasia revealed that mice immunized with DLnano_3BVE_HA_CA09 but not with DLmono_HA_CA09 were protected from lung pathology, including the observations of eosinophilic necrotic deposits within the alveolar spaces and or thickening of alveolar septa, associated with influenza infection (Figure 6g; Figure S6d, Supporting Information). The lethal challenge study illustrated that the DLnanovaccine could confer significant functional advantages in an infectious disease model.

## 3 Discussion

Development of vaccines can be a challenging endeavor due to poor immunogenicity of certain vaccine antigens, which results in the need to increase the number of required vaccinations, dose per vaccination, and the required interval for patients to complete the vaccine regime. Particulate vaccine formulations can help boost immunogenicity but can be slow to develop on a large scale due to manufacturing complexities. Synthetic nucleic‐acid‐based methods for the delivery of vaccine antigens have shown great promises, as they are often produced at significantly lower costs than their protein counterparts, can be manufactured to scale and bypass complex processes of assembly,^49^ offer superior safety profile,^50^ and demonstrate remarkable thermostability to allow for extended shelf‐lives.^51^

In this study, through the use of computational modeling and biophysical characterization, we engineered multimeric forms of HIV and influenza antigens that folded properly in vitro and displayed the desired antigenic profiles. We showed that these designer nanovaccines can assemble in vivo when delivered using synthetic DNA and adaptive EP, through direct evidence from pseudo‐native PAGE analysis (Figure 1f) and transmission electron microscopy (Figure 1h,i), and indirect evidence of binding of murine MBL to in vivo produced DLnano_LS_GT8 but not to DLmono_GT8 (Figure 1g). The in vivo nanoparticle assembly resulted in improved antigen trafficking and induction of potent adaptive immune responses, including rapid seroconversion, higher binding, and functional HAI antibody titers yet with significant dose sparing. Enhanced antibody responses were also induced when DLnanovaccine was administered via ID DNA vaccination, a newer and clinically important route of DNA vaccination.^37^, ^40^ Importantly, enhanced immune responses induced by DLnanovaccines also conferred functional advantages. The DLnanovaccines were more efficient at driving HAI, CD8+ T‐cell responses, and ultimately generating protection to animals from intranasal influenza challenge. DNA vaccine approach can effectively synergize with structure‐guided protein engineering to quickly produce in vivo designer nanovaccine constructs for rapid evaluation.

This work interrogated factors that might contribute to the enhanced adaptive immune responses of DLnanovaccines. Homogeneous in vitro assembly of these computationally designed DLnanovaccines is important for their downstream success, as poorly assembled DNA‐encoded LS_HA_CA09 did not elicit similarly potent immune responses (Figure S5f, Supporting Information). Homogeneous in vitro assembly will likely help increase the fraction of more fully assembled nanoparticles in vivo, contributing to the overall immunogenicity of the vaccine. It is in theory possible that the improved immunogenicity described here can be attributed to differences in levels of antigen expression. However, two observations suggest that antigen expression is not solely responsible for improved responses. First, we showed that DLnano_LS_GT8 induced stronger humoral responses than DLmono_GT8 in BALB/c mice at less than one‐tenth of the dose (Figure 2f). Second, while DLnano_PfV_GT8 expressed at higher levels in vivo than DLnano_3BVE_GT8 (Figure 4d), DLnano_PfV_GT8 and DLnano_3BVE_GT8 induced similar antibody titers and T cell responses at 25 µg dose (Figure 4e,f). The exact contribution of nanoparticle assembly, expression, and valency for the induction of optimal immune responses will require further investigation.

When DNA‐launched nanovaccines were compared to recombinant protein nanovaccines, widely considered as an extremely potent vaccine formulation in terms of induction of antibody responses,^52^ we observed DLnanovaccines induced comparable humoral responses to recombinant protein nanovaccines, but uniquely induced potent CD8+ T‐cell responses in an MBL‐complement‐independent manner. The observation that DLnanovaccines function independently of MBL‐complement pathway is likely of interest for clinical translation of such vaccines, as approximately 5–20% of human populations have MBL deficiency (plasma MBL < 100 ng mL^–1^).^53^, ^54^ The role of T cells in immune surveillance to mediate protection provides a strong rationale for exploring this unique property of DLnanovaccine,^55^ especially for such diseases as liver‐stage malaria,^56^ influenza for the elderlies,^57^, ^58^ and cancer.^59^ The unique ability for DLnano vaccination to induce CD8+ T‐cell responses may be related to its distinct mechanism of antigen uptake and presentation. Antigen‐presenting cells, such as macrophages, are known to migrate into the site of EPto scavenge antigens expressed through DNA cassettes associated with apoptotic cells.^49^ Prior studies observed that co‐delivery of DNA vaccines with proapoptotic‐mutated Caspase 2 or Fas significantly increased both CD4+ and CD8+ T‐cell responses to the vaccine antigens.^60^, ^61^ Such distinct mechanism of antigen processing might lead to more efficient cross‐presentation to the MHC class I pathway. Additionally, APCs including DCs and macrophages may also be directly transfected with the inoculated DNA cassettes in vivo,^62^, ^63^ and the two mechanisms may be synergistic in the induction of CD8+ T‐cell immunity. Our findings also demonstrated that DLnanovaccine could improve induced CD8+ T‐cell responses by eightfold to tenfold relative to their monomeric counterparts. Given that DNAvaccines can already induce CD8+ T‐cell responses in patients to cause histopathological regression of HPV‐driven cervical dysplasia,^4^ the finding is relevant and whether DLnanovaccines can further improve the clinical response rates should be explored.

Importantly, significant dose sparing can be realized with DLnanovaccines. A dose of 1 µg of plasmid DNA, a dose at which we historically would not expect to observe robust seroconversion,^64^ was able to induce clear functional HAI titers in mice. Fewer immunizations of DLnanovaccine could induce the same, if not higher, titers of antibodies. Given recent advances in the EP technology has improved the potency and consistency of immune responses induced by DNA vaccines in patients,^4^, ^37^, ^38^, ^39^, ^63^, ^65^ it will be important to determine whether DLnanovaccines can also help to reduce doses used in the clinic and lower the number of clinical visits required for vaccination. These advances may have important implications for outbreak control, and for global deployment including of vaccinations in more resource limited settings.

It will be important to build on these initial studies to improve DLnanovaccines. For example, while it is known that cross‐linking of B‐cell receptors through multivalent antigen display can improve B‐cell responses,^15^ studies to examine the mechanisms for the improved CD8+ T‐cell responses for DLnanovaccines relative to their monomeric counterparts are also important. Due to the unique ability of DLnanovaccine to elicit strong CD8+ T‐cell immunity, new DLnanovaccines should be designed and evaluated to target diseases such as cancer and T‐cell dependent infectious diseases. The combined advantages of a simplified cost‐effective temperature‐stable platform, with the ability to retain in vivo structural integrity may be of value for the development of additional vaccines for HIV, influenza as well as other infectious diseases.

## 4 Conclusion

This work demonstrates that advances in synthetic DNA and adaptive electroporation technologies have allowed for in vivo assembly of complex computationally designed particulate nanovaccines to induce improved humoral and cellular responses, and to confer functional protective benefits in a survival study. As DNA can be rapidly manufactured to scale with low costs, it can be envisioned that computationally designed nanovaccines can be rapidly evaluated to expedite clinical translational and global deployment of various promising vaccine candidates.

## 5 Experimental Section

##### Study Design

The authors were informed by their prior findings that synthetic DNA and electroporation can be used to deliver in vivo biologics such as antibodies and enzymes, and sought to determine in this study whether more complex structures such as macromolecular nanoparticle assemblies can also be delivered by DNA/EP. Nanovaccines have historically been shown to induce more potent humoral responses than their monomeric counterparts but may be challenging to produce on a large scale. Therefore, a method to simplify the process by producing these nanovaccines in the hosts may be relevant. Sample sizes in the study were predetermined by power analyses with results from another set of the pilot studies. All the collected datapoints were included in the final analyses except for a single guinea pig in the DLmono_GT8 group that showed preexisting antibody titers to GT8. All data were collected in at least technical duplicates, and except for the guinea pig and the challenge experiment, all findings were replicated successfully at least once in the study. Animals were randomly allocated to cages at the initiation of the study and were not further randomized. Data collection and analyses were not blinded. Detailed sample sizes can be found in the figure legends and statistical tests performed can be found in section Statistics.

##### Structure Modeling and Design of 3BVE, Ferritin, LS, PfV, and Flu Nanoparticles

The nanoparticle structures for ferritin (PDB ID: 3BVE), LS (PDB ID: 1HQK), and PfV (2E0Z) were used to seed the modeling simulations. The structure of eOD‐GT8 (PDB ID: 5IDL) and HA1 (PDB ID: 3GBN) was used to decorate the nanoparticles. N‐linked glycans with missing density were added using glycan modeling modules of Rosetta.^66^ A new algorithm (simpleNanoparticleModeling) was written in the Molecular Software Library.^67^ Briefly, the appropriate number of immunogens were aligned at the surface of the nanoparticle using coordinate frames constructed by 3 C‐α atoms of the terminal positions of each protein. Immunogens were then tilted by random rotations around the *x* and *y* axes up to 30° for the first three‐fourth of the simulation and up to 75° for the last one‐fourth of the simulation, with a 120° rotation allowed for the *z* axis. The immunogens were also translated by 10–200 Å along an axis projected away from the nanoparticle surface. Clashes were detected at each iterations and the models with the lowest number of clashes at each translation were written out as a potential structural model. The models were manually inspected and utilized to construct linkers as glycine‐serine repeats using 30 Å per 9 linker residues as a guide. The sequence of the HA isolate H1 NC99 (A/New Caledonia/30/1999 (H1N1)) from residues 65‐276 was used to construct the flu nanoparticle.

##### DNA Design and Plasmid Synthesis

Protein sequences for IgE Leader Sequence and eOD‐GT8‐60mer were as previously reported.^34^, ^68^ Protein sequences for 3BVE‐ferritin, PfV, and HA_CA09 were obtained from UniProt (accession numbers: Q9ZLI1, I6U7J4, and C3W5 ×2). Protein sequence for HA1_NC99 was obtained from GenBank (accession number: AY289929.1). DNA encoding protein sequences were codon and RNA optimized as previously described.^34^ The optimized transgenes were synthesized de novo (GenScript, Piscataway, NJ) and cloned into a modified pVAX‐1 backbone under the control of the human CMV promoter and bovine growth hormone polyadenylation signal. All the plasmid maxi‐preps were produced commercially (GenScript; Aldevron, Fargo, ND), with low endotoxin level (<0.005E U µg^–1^).

##### Production of His‐Tagged GT8‐Monomer and Recombinant Protein DLnanos

Expi293F cells were transfected with pVAX plasmid vector carrying the DLnano or His‐Tagged GT8‐monomer transgene with PEI/OPTI‐MEM and harvested 6 days post‐transfection. Transfection supernatant was first purified with affinity chromatography using the AKTA pure 25 system and an IMAC Nickel column (for His‐tagged GT8) and gravity flow columns filled with GNL Lectin beads (for DLnanos). The eluate fractions from the affinity purification were pooled, concentrated, and dialyzed into 1× PBS buffer before being loaded onto the SEC column and then purified with SEC, for which the Superdex 75 10/300 GL column was used to purify His‐tagged GT8‐monomer and the Superose 6 Increase 10/300 GL column was used for DLnanos (run at 0.5 mL min^–1^). Identified eluate fractions were then collected and concentrated to 1 mg mL^–1^ in PBS.

##### Immunization

All animal experiments were carried out in accordance with animal protocols 1127760 and 112782 approved by the Wistar Institute Institutional Animal Care and Use Committee (IACUC, Philadelphia, PA). For DNA‐based immunization, 6–8 week old female C57BL/6, BALB/c, and CD1 mice or 6–8 week old male BALB/c mice purchased from Jackson Laboratory or Charles River Laboratories were immunized one to three times (3 weeks apart) with DLmono_GT8, DLmono_HA_NC99, DLmono_HA_CA09, DNA‐encoded LS_HA_CA09, DL_GT8_IMX313P or DLnano_LS_GT8, DLnano_CD4MutLS_GT8, DLnano_3BVE_GT8, DLnano_PfV_GT8, DLnano_LS_HA_NC99, and DLnano_3BVE_HA_CA09 via intramuscular injections into the tibialis anterior muscles (over two sites), followed by intramuscular electroporation with the CELLECTRA 3P device (Inovio Pharmaceuticals). For electroporation, two sets of two pulses (at 0.1 Amps) were delivered. Each set of two pulses lasts 52 ms with a 1 s delay. For all DNA‐encoded GT8‐based immunizations (except for dosing studies), 25 µg of plasmid DNA was used, a standard DNA dose as in prior study.^69^ For the control experiment to assess the importance of antigen decoration on nanoparticle, BALB/c mice were immunized with 1:1 co‐formulated (25 µg each) DLmono_GT8 with pVAX, DLmono_GT8 with DLnano_LS (core), and DLnano_LS_GT8 with pVAX and followed for 7 d.p.i. for seroconversion. For all DNA‐encoded HA‐based immunizations, doses of 1 µg were used for each immunization for studies of humoral responses and 10 µg for studies of cellular responses. MBL knockout mice (B6.129S4‐Mbl1tm1Kata Mbl2tm1Kata/J) and CR2 knockout mice (B6.129S7(NOD)‐Cr2tm1Hmo/J) purchased from Jackson Laboratory were immunized in the same manner.

For protein‐based immunization, 6–8 week old female C57BL/6, MBL knockout and CR2 knockout mice were immunized subcutaneously over two sites with a high dose of 10 µg of recombinant eOD‐GT8‐60mer protein in 50 µL co‐formulated with 50 µL Sigma adjuvant system (Sigma‐Aldrich); the protein dose was 2.7 times higher than a prior study.^26^

Female Hartley guinea pigs (8–10 weeks old) purchased from Charles River Laboratories (Wilmington, MA) were group housed and handled at Acculab (San Diego, CA) with ad libitum access to food and water according to IACUC protocol CalMI2‐043. Following acclimation, each guinea pig was given a single immunization of 50 µg of DLnano_LS_GT8 or DLmono_GT8 over two sites on the flank followed by ID EP with CELLECTRA 3P device. The animals were then bled at the indicated timepoints for humoral analyses.

##### ELISA—GT8‐Binding ELISA

Corning 96‐well half area plates were coated at room temperature for 6 h with 1 μg mL^–1^ MonoRab anti‐His antibody (GenScript), followed by overnight blocking with solution containing 1× PBS, 5% skim milk, 10% goat serum, 1% BSA, 1% FBS, and 0.2% Tween‐20. The plates were then incubated with 2 μg mL^–1^ of his‐tagged GT8‐monomer at room temperature for 2 h, followed by addition of mice sera serially diluted with PBS with 1% FBS and 0.1% Tween and incubation at 37 °C for 2 h. The plates were then incubated at room temperature for 1 h with Peroxidase AffiniPure Goat Anti‐Mouse IgG, Fcγ fragment specific at 1:5000 dilution (Jackson ImmunoResearch) or AffiniPure Goat Anti‐Mouse IgM, μ chain specific, (Jackson ImmunoResearch) at 1:5000 dilution followed by addition of TMB substrates (Thermo Fisher Scientific) and then quenched with 1 M H_2_SO_4_. Absorbance at 450 and 570 nm were recorded with BioTEK plate reader. Endpoint titer is defined as the highest dilution at which the OD of the post‐immune sera exceeds the cutoff (mean OD of naïve animals plus standard deviations of the OD in the naïve sera multiplied with standard deviation multiplier *f* at the 99% confidence level).

##### ELISA—VRC01 Competition ELISA

The plates were coated, blocked, and followed by addition with GT8‐his as described in the last section. Serially diluted mice sera were then incubated with the plates at 37 °C for 1 h, followed by addition of purified VRC01 antibody (NIH AIDS Reagent) for an additional 1 h at room temperature. The plates were then incubated with anti‐human Fc (cross‐adsorbed against rabbits and mice) (Jackson ImmunoResearch) at 1:10000 dilution for 1 h, followed by addition of TMB substrate for detection. Absorbance at 450 and 570 nm were recorded with BioTEK plate reader.

##### ELISA—MBL‐Binding ELISA

The plates were coated with 5 µg mL^–1^ recombinant mouse MBL protein (R&D System) in 0.1 M CaCl_2_ at room temperature for 6 h, followed by blocking with 1% BSA in 0.1 M CaCl_2_ in PBS overnight at 4 °C. Transfection supernatant or muscle homogenates containing DLmono_GT8 or DLnano_LS_GT8 were then added to the plates for 2 h incubation at 37 °C, followed by week 5 sera of BALB/c mice previously immunized twice with 25 µg DLnano_LS_GT8. The plates were then incubated with anti‐mouse IgG H+L (cross‐adsorbed against human) HRP (Jackson ImmunoResearch) at 1:10000 dilution, followed by addition of TMB substrates. Absorbance at 450 and 570 nm was recorded with BioTEK plate reader.

##### ELISA—VRC01‐Binding ELISA

ELISA format as described in the section ELISA—MBL‐Binding ELISA except that the recombinant MBL used in the coating step is replaced by 5 µg mL^–1^ of VRC01 (NIH AIDS Reagent). Absorbance at 450 and 570 nm was recorded with BioTEK plate reader.

##### ELISA—Antigenic Profile Characterization of Designed GT8‐Nanovaccines

Corning half‐area 96‐well plates were coated with 2 µg mL^–1^ of GT8‐monomer, or 3BVE_GT8‐24mer, eOD‐GT8‐60mer, CD4Mut_LS_GT8‐60mer, and PfV_GT8‐180mer at 4 °C overnight. The plates were then blocked with the buffer as described in the GT8‐binding ELISA section for 2 h at room temperature, followed by incubation with serially diluted VRC01 at room temperature for 2 h. The plates were then incubated with anti‐human Fc (cross‐adsorbed against rabbits and mice) (Jackson ImmunoResearch) at 1:10000 dilution for 1 h, followed by addition of TMB substrate for detection. Absorbance at 450 and 570 nm was recorded with BioTEK plate reader.

##### ELISA—HA‐Binding ELISA

Corning 96‐well half area plates were coated at 4 °C overnight with 2 µg mL^–1^ of recombinant HA(ΔTM)(H1N1/A/New Caledonia/20/1999) or HA(ΔTM)(A/California/04/2009)(H1N1) (Immune Technology), and blocked at room temperature for 2 h with the buffer as described in the GT8‐binding ELISA section. The plates were subsequently incubated with serially diluted mouse sera in PBS with 1% FBS and 0.1% Tween at 37 °C for 2 h, followed by 1 h incubation with anti‐mouse IgG H+L HRP (Bethyl) at 1:20000 dilution at room temperature and development with the use of TMB substrate. Absorbance at 450 and 570 nm was recorded with BioTEK plate reader.

##### HAI Assay

Mice sera were treated with receptor‐destroying enzyme (RDE, 1:3 ratio) at 37 °C overnight for 18–20 h followed by complement and enzyme inactivation at 56 °C for 45 min. RDE‐treated sera were subsequently cross‐adsorbed with 10% rooster red blood cells (Lampire Biologicals) in PBS at 4 °C for 1 h. The cross‐adsorbed sera were then serially diluted with PBS in a 96‐well V‐bottom microtiter plates (Corning). Four hemagglutinating doses (HAD) of A/Solomon Islands/03/06 virus, A/New Caledonia/20/99, or A/California/07/2009 (BEI) were added to each well and the serum–virus mixture was incubated at room temperature for 1 h and then incubated with 50 µL 0.5% v/v rooster red blood cells in 0.9% saline for 30 min at room temperature. The HAI antibody titer was scored with the dot method, and the reciprocal of the highest dilution that did not exhibit agglutination of the rooster red blood cells was recorded.

##### Immunofluorescence

For lymph node staining, 7 days after BALB/c mice were immunized with 80 µg DNA co‐formulated with 12 U hyaluronidase (Sigma) encoding GT8‐monomer or DLnano_LS_GT8, tibialis anterior muscles of the mice were injected with 5 µg of anti‐mouse CD35 BV421 (BD Bioscience) for in situ labeling of follicular dendritic cells 16 h prior to harvest. Ipsilateral iliac lymph nodes from the mice were harvested the next day and preserved in O.C.T. medium (Fisher) for cryosectioning. The sections were fixed with 4% paraformaldehyde and blocked in 3% BSA/PBS for 1 h at room temperature, followed by overnight staining with 6 µg mL^–1^ VRC01. The sections were then washed and stained with anti‐human Alexa Fluor 488 antibody and imaged with Leica SP5 confocal microscopes.

For muscle staining, 4 days after BALB/c mice were immunized with 80 µg DNA encoding GT8‐monomer, DLnano_LS_GT8, DLnano_3BVE_GT8, or DLnano_PfV_GT8 co‐formulated with 12 U hyaluronidase in the tibialis anterior muscles of the mice were harvested the and preserved in 4% PFA/PBS for 2 h at room temperature and then stored overnight in 70% EtOH/H_2_O at 4 °C. The tissues were then serially dehydrated and blocked in 3% BSA/PBS for 1 h at room temperature, followed by overnight staining with 6 µg mL^–1^ VRC01. The sections were then washed, and stained with anti‐human Alexa Fluor 488 antibody, counterstained with 0.5 µg mL^–1^ DAPI and imaged with Leica SP5 confocal microscopes.

For transfected cells, HEK293T cells were cultured in poly‐lysine‐coated glass chambers overnight, and then transfected with DNA‐encoding GT8‐monomer or eOD‐GT8‐60mer with GeneJammer (Agilent). The cells were harvested 48 h post‐transfection, fixed with 4% paraformaldehyde, permeabilized with 0.5% Triton X‐100/PBS, blocked, and stained as in the section describing muscle immunofluorescence staining.

##### Immunohistochemistry

For immunohistochemistry staining of muscle sections, BALB/C mice were immunized with 80 µg DLmono_GT8 or DLnano_LS_GT8 co‐formulated with 12 U hyaluronidase (Sigma). Transfected muscles were harvested 7 days post‐immunization, cryosectioned, fixed, permeabilized, and blocked as described in the Immunofluorescence section. The muscle sections were then stained with goat anti‐mouse MBL at 1:200 dilution in 1% BSA/PBS (R&D System) overnight, and then with secondary Rabbit anti‐goat (H+L) HRP conjugated at 1:500 dilution (BioRad) and DAB substrates for development.

##### Electron Microscopy—Transmission EM of Muscles

Tibialis anterior muscles from BALB/c mice immunized with 80 µg DLmono_GT8 or DLnano_LS_GT8 co‐formulated with 12 U hyaluronidase were collected 7 d.p.i. The muscles were then fixed in 2.5% glutaraldehyde, serially dehydrated in acetone/ethanol solvents, and then embedded in epoxy and LR white resin. The resin was then sectioned to a thickness of 70 nm and deposited onto a metal grid, blocked overnight in 3% BSA/PBS, followed by staining with 60 µg mL^–1^ VRC01 (diluted in 3% BSA/PBS) overnight, and with 1:200 anti‐human 6 nm gold nanoparticles (Jackson ImmunoResearch) for 1 h. The sections were then washed with 0.1% Tween in PBS, and water, followed by post‐staining fixation with 2.5% glutaraldehyde in PBS for 5 min at room temperature followed by staining with 2% uranyl acetate for 1 h. The grids were subsequently imaged with JEOL JEM 1010 transmission electron microscope. For quantitative analyses, total number of gold‐labeled clusters and order of each cluster were manually counted. Frequency of a cluster of a particular order in a field of view was normalized relative to the total number of clusters observed.

##### Negative Stain EM of Purified Nanoparticles

The nanoparticles were produced in Expi293 cells, purified using Agarose‐bound lectin beads (Agarose Galanthus Nivalis Lectin, Vector Laboratories) followed by SEC (GE Healthcare) using the Superose 6 Increase 10/300 GL column. The proteins were further dialyzed into Tris‐buffered saline (TBS). A total of 3 µL of purified proteins was adsorbed onto glow discharged carbon‐coated Cu400 EM grids. The grids were then stained with 3 µL of 2% uranyl acetate, blotted, and stained again with 3 µL of the stain followed by a final blot. Image collection and data processing was performed on a FEI Tecnai T12 microscope equipped with Oneview Gatan camera at 90 450× magnification at the camera and a pixel size of 1.66 Å.

##### ELISpot Assay

Spleens from immunized mice were collected 5 weeks after the first immunization (2 weeks after the second immunization) and homogenized into single‐cell suspension with a tissue stomacher in 10% FBS/1% penicillin‐streptomycin in RPMI 1640. Red blood cells were subsequently lysed with ACK lysing buffer (Thermo Fisher Scientific) and percentage of viable cells were determined with Trypan Blue exclusion. A total of 200 000 cells were then plated in each well in the mouse IFNγ ELISpot plates (MabTech), followed by addition of peptide pools that span both the LS, GT8 or HA domains at 5 µg mL^–1^ of final concentration for each peptide (GenScript). The cells were then stimulated at 37 °C for 16–18 h, followed by development according to the manufacturer's instructions. Spots for each well were then imaged and counted with ImmunoSpot Macro Analyzer.

##### Intracellular Cytokine Staining

Single cell suspension from spleens of immunized animals was prepared as described in the previous section and stimulated with 5 µg mL^–1^ of peptides spanning both the LS, GT8 or HA domains (GenScript) for 5 h at 37 °C in the presence of 1:500 protein transport inhibitor (Thermo Fisher Scientific) and anti‐mouse CD107a‐FITC (Thermo Fisher Scientific). The cells were then incubated with violet fluorescent reactive (live/dead dye) for 10 min at room temperature, surface stains (anti‐mouse CD4 BV510, anti‐mouse CD8 APC‐Cy7, anti‐mouse CD62L BV711, and anti‐mouse CD44 AF700) (BD‐Biosciences) at room temperature for 30 min. The cells were then fixed and permeabilized according to the manufacturer's instructions for BD Cytoperm Cytofix kit and stained with intracellular stains anti‐mouse IL‐2 PE‐Cy7, anti‐mouse IFN‐γ APC, anti‐mouse CD3e PE‐Cy5, and anti‐mouse TNFα BV605 (BioLegend) at 4 °C for 1 h. The cells were subsequently analyzed with LSR II 18‐color flow cytometer.

##### Immunoblotting

Tibialis anterior muscles of immunized animals were harvested and homogenized in T‐PER extraction buffer (Thermo Fisher Scientific) and protease inhibitor (Roche). Muscle homogenates were subsequently concentrated 20× with Amicon Ultra 0.5 mL centrifugation kits with 3kDA cutoffs (Milipore Sigma) and protein concentrations were quantified with BCA assays (Thermo Fisher Scientific). For electrophoresis, 8 µL supernatants of Expi293F cells transfected with pVAX, DLmono_GT8, eOD‐GT8‐60mer, or 50 µg muscle homogenates from mice immunized with the 80 µg aforementioned constructs co‐formulated with 12 U hyaluronidase were loaded onto 4–12% SDS Bis‐Tris gel (SDS‐PAGE) or 3–8% Tris‐acetate gel (pseudo‐native PAGE) for electrophoresis. For SDS‐PAGE, all samples were reduced with heating of the samples in the presence of a reducing agent and LDS sample buffer (Thermo Fisher Scientific) at 70 °C for 10 min. For pseudo‐native PAGE, samples were only incubated with the LDS buffer at room temperature and loaded directly onto the 3–8% TA gel without boiling. Proteins were subsequently transferred to PVDF membrane from the gels and stained overnight at 4 °C with 3 µg mL^–1^ VRC01 and 1 µg mL^–1^ anti‐human GAPDH (for SDS‐PAGE only, Clone D4C6R, Cell Signaling) in Odyssey Blocking Buffer/PBS/0.1% Tween (LI‐COR Biosciences), and 1:10000 IRDye 800CW goat anti‐human IgG (LI‐COR Biosciences) in Odyssey Blocking Buffer/0.1% Tween/0.1% SDS at room temperature for 1 h, and then scanned with LI‐COR Odyssey CLx.

##### Determination of the Antigen‐Specific B‐Cells in Spleen

Recombinant 3BVE‐GT8 was labeled with FITC with the lightning link kits according to manufacturer's instructions (Expedon). Spleens were harvested 5 weeks after the second immunization of 25 µg of DLnaono_LS_GT8, DLmono_GT8 or from naïve mice. Single cells were then labeled with LIVE/DEAD dye ultraviolet reactive (Thermo Fisher Scientific) at room temperature for 10 min and incubated with mouse Fc‐Block (Clone 93, Thermo Fisher Scientific) at 1:200 dilution. Avi‐tagged GT8 was biotinylated and tetramerized with an excess of APC‐streptavidin (Thermo Fisher Scientific) as previously described.^27^ The cells were washed with PBS and incubated with 1:200 A488‐3BVE‐GT8 and 1:200 APC‐GT8‐tetramer at 4 °C for 30 min. Without being washed, the cells were incubated with 1:200 anti‐mIgD‐APC/Cy7 (BioLegend), anti‐mIgM‐BV711 (Thermo Fisher Scientific), anti‐mCD19‐PECy7 (BioLegend), and anti‐mIgG‐BV510 (BioLegend) in 1% FBS/PBS solution. The cells were then resuspended in 1× BDFix buffer and analyzed with LSR II 18‐color flow cytometer.

##### Lethal H1/A/California/07/09 Influenza Challenge

Six to eight week‐old female BALB/c mice (Jackson Laboratory) were immunized with 1 µg of pVAX vector, DLmono_HA_CA09, or DLnano_3BVE_HA_CA09 twice 3 weeks apart. The mice were subsequently transferred to BioQual, Inc. for challenge experiment. Thirty‐five days after the second immunization, the mice were intranasally inoculated with 10LD_50_ H1/A/California/07/09 in PBS. Weights of the mice were pre‐recorded prior to the challenge and daily after the challenge until 7 d.p.i., at which lungs from the mice were harvested and snap‐frozen for viral load assay by RT‐qPCR and histopathology by H&E staining. At any point, mice exhibiting more than 20% of weight loss as compared to baseline were euthanized (humane endpoint).

##### RT‐qPCR Assay for Viral Load Determination

The amounts of RNA copies per gram lung tissue were determined using a real‐time quantitative PCR (qPCR) assay. This assay utilized primers and a probe specifically designed to amplify and bind to a conserved region of the NP gene of influenza virus. The signal was compared to a known standard curve and calculated to give copies per gram tissue. Viral RNA was extracted from lung homogenates using MiniElute Virus Spin Kit (Qiagen). TAQMAN RT‐PCR kit (Applied Biosystems, Inc., Carlsbad, CA) was used for amplification of viral RNA in the presence of 600 nM primers (CAL‐1‐U: ATGGCGTCTCAAGGCACCAA and CAL‐1‐D: GCACATTTGGATGTAGAATCTC) and 140 nM probe (CAL‐1‐P: 6FAM‐CAGAGCATCTGTCGGAAGAATGATTG‐TAMRA) with the following thermocycler setting: 48 °C for 30 min, 95 °C for 10 min followed by 40 cycles of 95 °C for 15 s, and 1 min at 60 °C.

##### Statistics

Power analysis was performed with R based on the preliminary data to determine the smallest sample size that would allow to achieve a power of 0.9 with a pre‐set α‐value of 0.05. All statistical analyses were performed with PRISM V8.0 and R V3.5.1. Each individual data point was sampled independently. Two‐tailed Mann–Whitney rank tests were used to compare differences between groups. Bonferroni corrections were used when multiple comparisons were made.

## Conflict of Interest

Z.X., D.B.W., and D.W.K. have a pending patent US.62784318. M.C.W., P.D.F., K.S., E.S., K.E.B., and L.M.H. are employees of Inovio Pharmaceuticals and as such receive salary and benefits including ownership of stock and stock options from the company. K.M. receives grants and consulting fees from Inovio related to DNA vaccine development. D.B.W. has received grant funding, participates in industry collaborations, has received speaking honoraria, and has received fees for consulting, including serving on scientific review committees and board series. Remuneration received by D.B.W. includes direct payments, stock or stock options, and in the interest of disclosure, he notes potential conflicts associated with his work with Inovio and possible others.

## Author Contributions

Z.X., M.C.W., D.B.W., and D.W.K. conceptualized the project. Z.X., M.C.W., S.T.C.E., A.P.‐P., P.D.F., K.S., V.M.‐B., D.B.W., and D.W.K. planned the experiments. Z.X., M.C.W., N.C., S.W., P.X., E.T.‐R., X.Z., R.A.P., P.D.F., S.G., H.A., and E.S. conducted the experiments. K.S., K.E.B., L.M.H., K.M., V.M.‐B., S.M., and W.R.S contributed crucial reagents or equipment. Z.X., D.B.W., and D.W.K analyzed the data. Z.X., D.B.W., and D.W.K. wrote the article.

## Supporting information

Supporting Information
