# Supplementary material for: In Vivo Assembly of Nanoparticles Achieved through Synergy of Structure‐Based Protein Engineering and Synthetic DNA Generates Enhanced Adaptive Immunity
Source: Adv Sci (Weinh). 2020 Feb 27;7(8):1902802. doi: 10.1002/advs.201902802 (PMC7175333; doi:10.1002/advs.201902802)
Supplement: Supplementary file 1 — Supporting Information [file ADVS-7-1902802-s001.pdf]

Copyright WILEY-VCH Verlag GmbH & Co. KGaA, 69469 Weinheim, Germany, 2020.

ADVANCED SCIENCE
Open Access

Supporting Information

for *Adv. Sci.,* DOI: 10.1002/advs.201902802

In Vivo Assembly of Nanoparticles Achieved through Synergy of Structure-Based Protein Engineering and Synthetic DNA Generates Enhanced Adaptive Immunity

Ziyang Xu, Megan C. Wise, Neethu Chokkalingam, Susanne Walker, Edgar Tello-Ruiz, Sarah T. C. Elliott, Alfredo Perales-Puchalt, Peng Xiao, Xizhou Zhu, Ruth A. Pumroy, Paul D. Fisher, Katherine Schultheis, Eric Schade, Sergey Menis, Stacy Guzman, Hanne Andersen, Kate E. Broderick, Laurent M. Humeau, Kar Muthumani, Vera Moiseenkova-Bell, William R. Schief, David B. Weiner, and Daniel W. Kulp**

Copyright WILEY-VCH Verlag GmbH & Co. KGaA, 69469 Weinheim, Germany, 2018.

Supporting Information

Title: In Vivo Assembly of Nanoparticles achieved through Synergy of Structure-based Protein Engineering and Synthetic DNA Generates Enhanced Adaptive Immunity

Ziyang Xu, Megan C. Wise, Neethu Chokkalingam, Susanne Walker, Edgar Tello-Ruiz, Sarah T.C. Elliott, Alfredo Perales-Puchalt, Peng Xiao, Xizhou Zhu, Ruth A. Pumroy, Paul D. Fisher, Katherine Schultheis, Eric Schade, Sergey Menis, Stacy Guzman, Hanne Andersen, Kate E. Broderick, Laurent M. Humeau, Kar Muthumani, Vera Moiseenkova-Bell, William R. Schief, David B. Weiner* and Daniel W. Kulp*

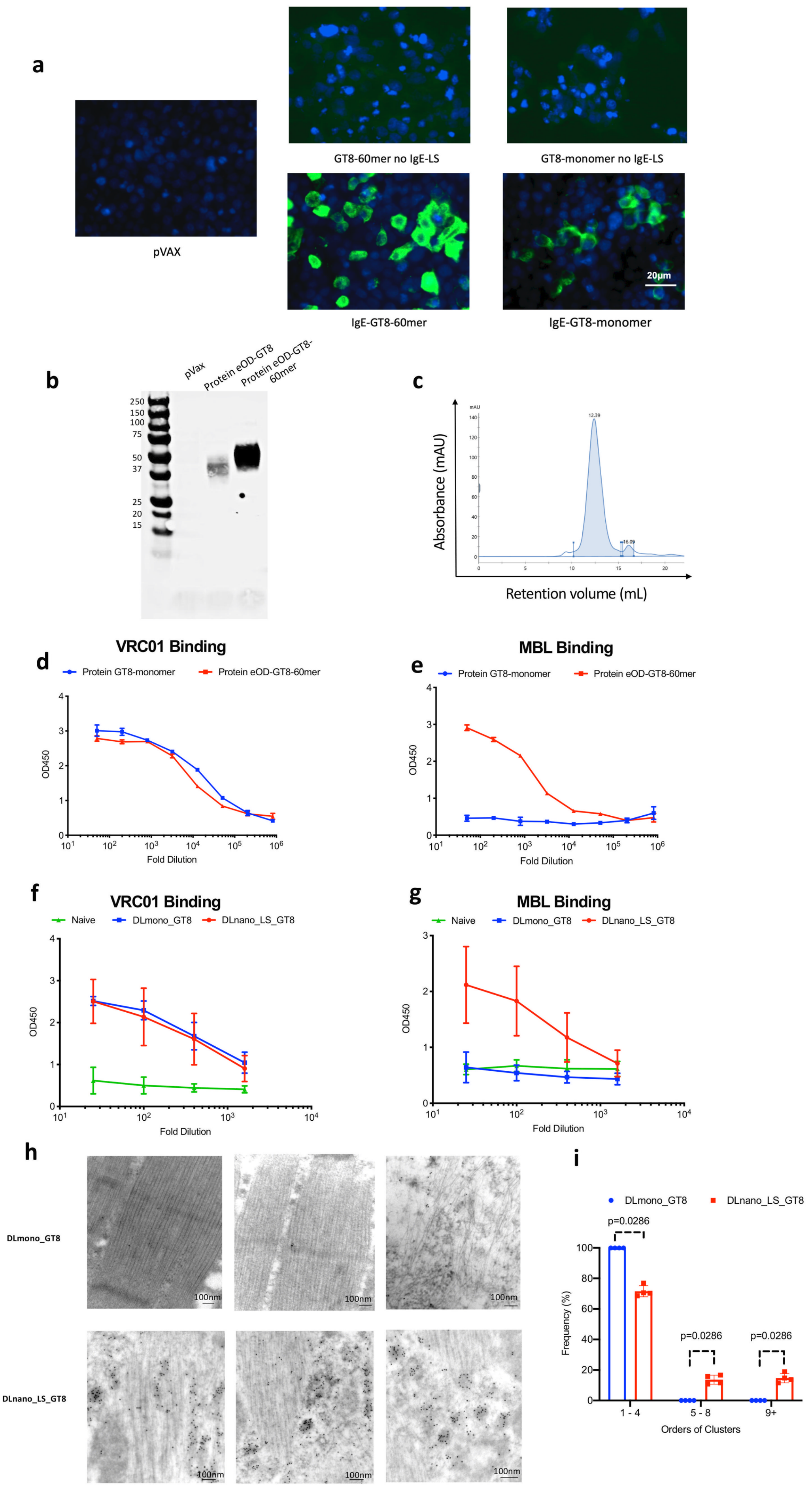
a
GT8-60mer no IgE-LS
GT8-monomer no IgE-LS
pVAX
IgE-GT8-60mer
IgE-GT8-monomer
20µm
b
pVax
Protein eOD-GT8
Protein eOD-GT8-60mer
250
150
100
75
50
37
25
20
15
c
Absorbance (mAU)
Retention volume (mL)
12.39
d
VRC01 Binding
Protein GT8-monomer
Protein eOD-GT8-60mer
OD450
Fold Dilution
e
MBL Binding
Protein GT8-monomer
Protein eOD-GT8-60mer
OD450
Fold Dilution
f
VRC01 Binding
Naive
DLmono_GT8
DLnano_LS_GT8
OD450
Fold Dilution
g
MBL Binding
Naive
DLmono_GT8
DLnano_LS_GT8
OD450
Fold Dilution
h
DLmono_GT8
DLnano_LS_GT8
100nm
i
DLmono_GT8
DLnano_LS_GT8
p=0.0286
Frequency (%)
1 - 4
5 - 8
9+
Orders of Clusters

Supplemental Figure 1. *In vitro* expression of protein eOD-GT8-60mer and GT8-monomer and *in vivo* expression and assembly of DLnano_LS_GT8 and DLmono_GT8. **a.** Immunofluorescence analyses of intracellular expression of protein eOD-GT8-monomer and -60mer with or without IgE leader sequence in transfected HEK293T cells as determined by staining with VRC01 (green) and DAPI (blue) staining. **b.** Reducing SDS-PAGE analysis of Expi293F transfection supernatants of pVAX backbone plasmid, protein GT8-monomer, protein eOD-GT8-60mer. **c**. SEC trace of lectin column purified Expi293F transfection supernatant of eOD-GT8-60mer. **d-e.** Binding of *in vitro* produced protein eOD-GT8-monomer and eOD-GT8-60mer to VRC01 (**d.**) and MBL (**e.**) in ELISA assays. **f-g.** Binding of *in vivo* expressed DLmono_GT8 and DLnano_LS_GT8 seven d.p.i to VRC01 (**f.**) and MBL (**g.**) in the ELISA assays. **h.** Additional TEM images of muscle sections from mice injected with DLmono_GT8 and DLnano_LS_GT8. **i.** Quantitative determination of the frequencies of clusters of different orders in the TEM images; *, $p<0.05$. 80ug of plasmid DNA used *in vivo* for panels **f** through **i**.

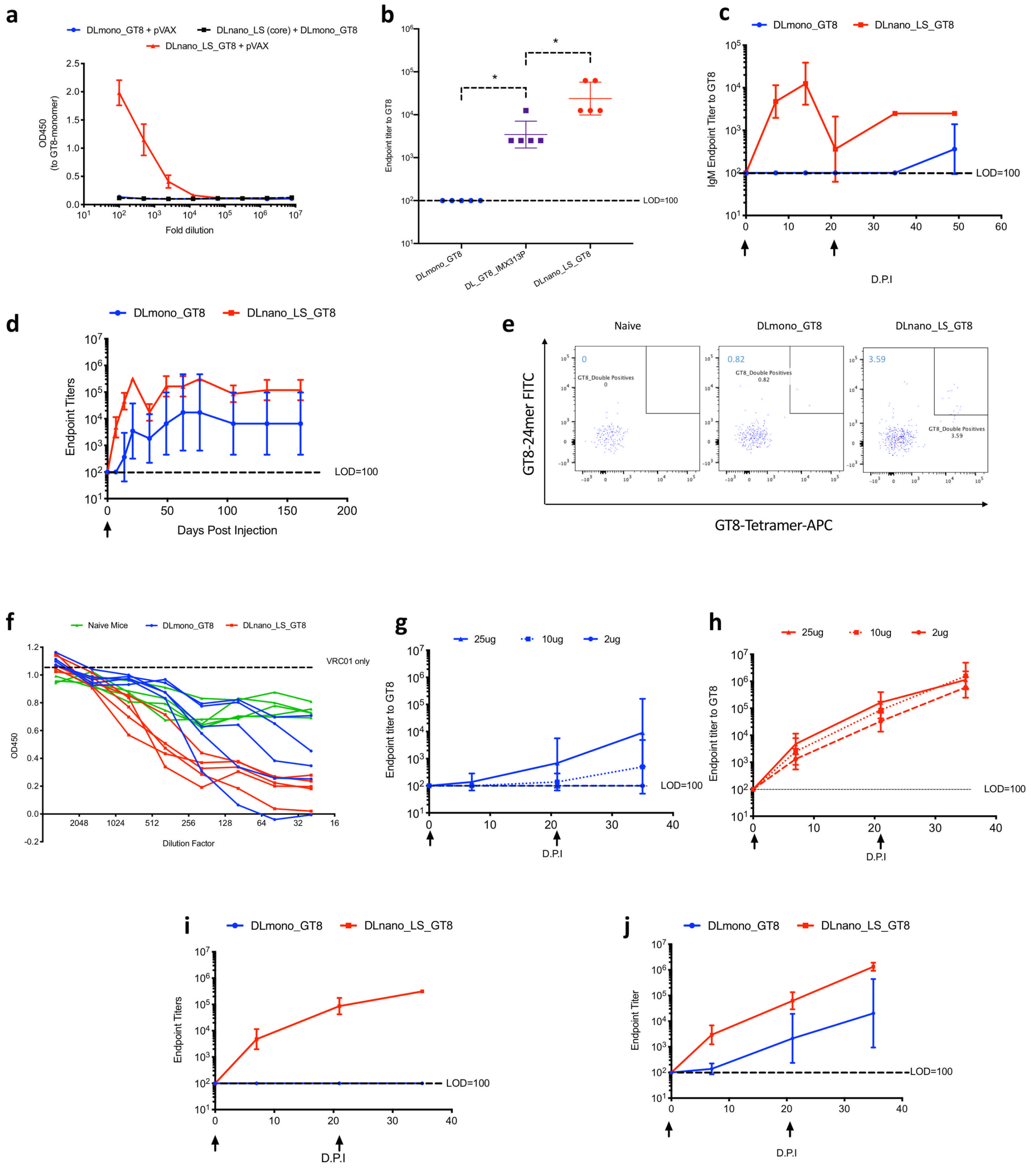

Supplemental Figure 2. Humoral responses induced by DLnano_LS_GT8 versus DLmono_GT8 vaccination in female BALB/c, C57BL/6 and CD1 mice. **a.** ELISA binding against monomeric GT8 using serum from BALB/c immunized with 1:1 ratio (25ug each) of DLmono_GT8 with pVAX backbone plasmid, DLmono_GT8 with DLnano_LS(core) or DLnano_LS_GT8 with pVAX backbone seven d.p.i. **b.** Endpoint titers at seven d.p.i in BALB/c mice immunized with DLmono_GT8, DL_GT8_IMX313P, or DLnano_LS_GT8. **c.** IgM endpoint titers to GT8 over time in BALB/c mice immunized with two doses of DLmono_GT8, or DLnano_LS_GT8. **d.** Endpoint titers to GT8 over time using serum from BALB/c receiving single immunizations of DLmono_GT8 or DLnano_LS_GT8. **e.** Flow plot demonstration of

gating of antigen-specific GT8-Tetramer-APC+ GT8-24mer-FITC+ CD19+IgM-IgD-IgG+ B-cells in the spleens of BALB/c mice immunized with two doses of DLmono_GT8 or DLnano_LS_GT8 five weeks post the second immunization **f.** ELISA data showing competition of VRC01 binding at its corresponding EC_{70} concentration to GT8 by week five post-immune sera from mice immunized with two doses of DLmono_GT8 or DLnano_LS_GT8. **g.** Endpoint titers to GT8 using serum for BALB/c receiving two immunizations of varying doses of DLmono_GT8. **h.** Endpoint titers to GT8 using serum from BALB/c mice receiving two immunizations of varying doses of DLnano_LS_GT8. **i**. Humoral responses in C57BL/6 mice immunized with two doses of DLmono_GT8 or DLnano_LS_GT8. **j.** Humoral responses in CD1 mice immunized with two doses of DLnano_LS_GT8 or DLmono_GT8. 25ug of plasmid DNA used in these experiments unless otherwise specified. n=5 for BALB/c and C57BL/6 mice, n=10 for CD1 mice; each line represents an animal; error bar represents standard deviation; arrow below the plot represents an immunization; *, $p<0.05$.

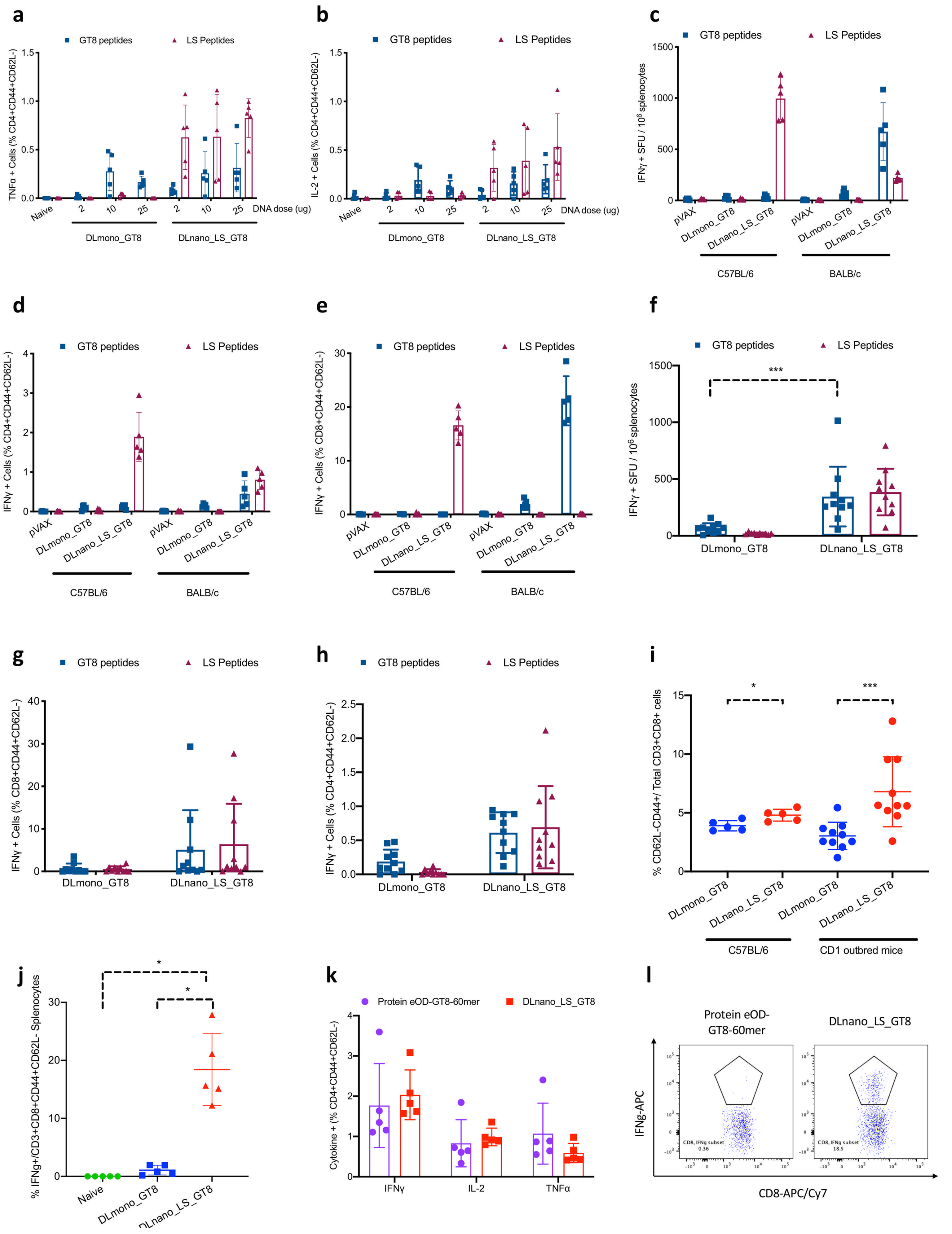

Supplemental Figure 3. Generalizability of improved cellular responses of DLnano_LS_GT8 in BALB/c, C57BL/6 and CD1 mice strains; comparison of induced CD8+ T-cell responses by protein eOD-GT8-60mer versus DLnano_LS_GT8 in C57BL/6 mice. **a** and **b**. Frequencies of TNFα and IL-2 expressing CD4+ effector memory T-cells specific to either the LS and GT8 domains in female BALB/c mice immunized twice with DLmono_GT8 or DLnano_LS_GT8.

c. Comparisons of total cellular responses to the LS and GT8 domains as assessed by IFN-γ ELISpot assay in female C57BL/6 versus BALB/c mice. **d.** and **e.** Comparison of CD4+ (**d.**) and CD8+ (**e.**) effector memory T-cell responses to the LS and GT8 domains in female C57BL/6 and BALB/c mice. **f**-**h**. Overall T-cell (**f**), CD4+ effector memory (**g**), and CD8+ effector memory (**h**) T-cell responses in female CD1 mice immunized with two doses of DLnano_LS_GT8 as compared to DLmono_GT8. **i**. Frequencies of effector memory CD8+ T-cells in female C57BL/6 and CD1 mice immunized twice with DLnano_LS_GT8 and DLmono_GT8. **j.** Comparison of frequencies of GT8-specific CD8+ T-cell responses induced by two immunizations of DLnano_LS_GT8 versus DLmono_GT8 in male BALB/c mice. **k.** CD4+ effector memory T-cell responses induced by protein eOD-GT8-60mer and DLnano_LS_GT8 in C57BL/6 mice as determined by ICS. **l.** Flow plot demonstrating induction of CD8+ effector memory T-cell responses by DLnano_LS_GT8 in comparison to protein eOD-GT8-60mer in C57BL/6 mice as determined. 25ug plasmid DNA and 10ug recombinant protein used in the figure unless otherwise specified. n=5 for BALB/c and C57BL/6 mice, n=10 for CD1 mice; each dot represents an animal; error bar represents standard deviation; two-tailed Mann-Whitney Rank Test used to compare groups; *, $p<0.05$; ***, $p<0.0005$.

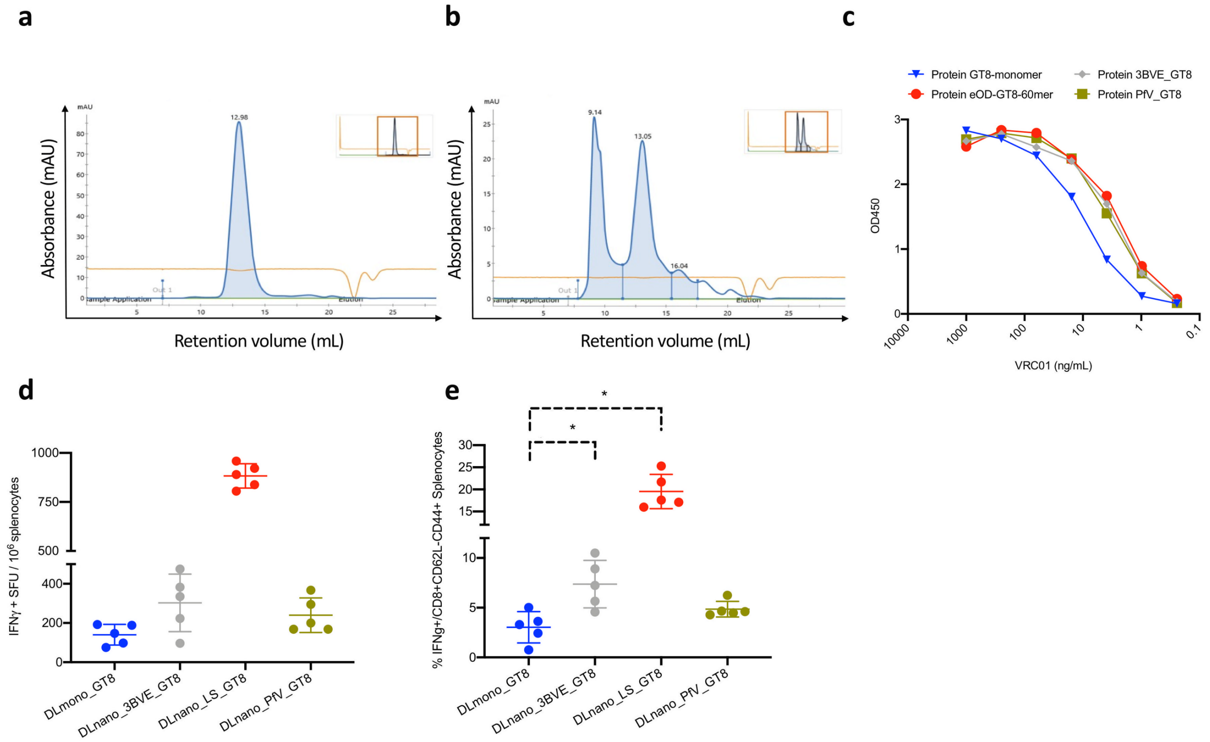

Supplemental Figure. 4. Characterization of biophysical profiles and immune responses induced by newly designed DLnano GT8-vaccines with alternative scaffolds. **a.** SEC trace of recombinantly produced designed 3BVE-GT8. **b.** SEC trace of designed PfV_GT8 immunogen shows partial assembly into the 180-mer form. **c.** Binding of *in vitro* produced protein GT8-monomer, 3BVE_GT8, eOD-GT8-60mer and PfV_GT8 to VRC01 by ELISA. **d** and **e.** Effector memory T-cell responses to GT8 domain in BALB/c mice immunized with two doses of 25ug DLmono_GT8, DLnano_3BVE_GT8, DLnano_LS_GT8 and DLnano_PfV_GT8 by IFNγ ELIspots (**d**) and ICS for CD8+ T-cells (**e**). n=5 per group; each dot represents an animal; error bar represents standard deviation; two-tailed Mann-Whitney Rank Test used to compare groups; p-values were adjusted for multiple comparison where appropriate; *, $p<0.05$.

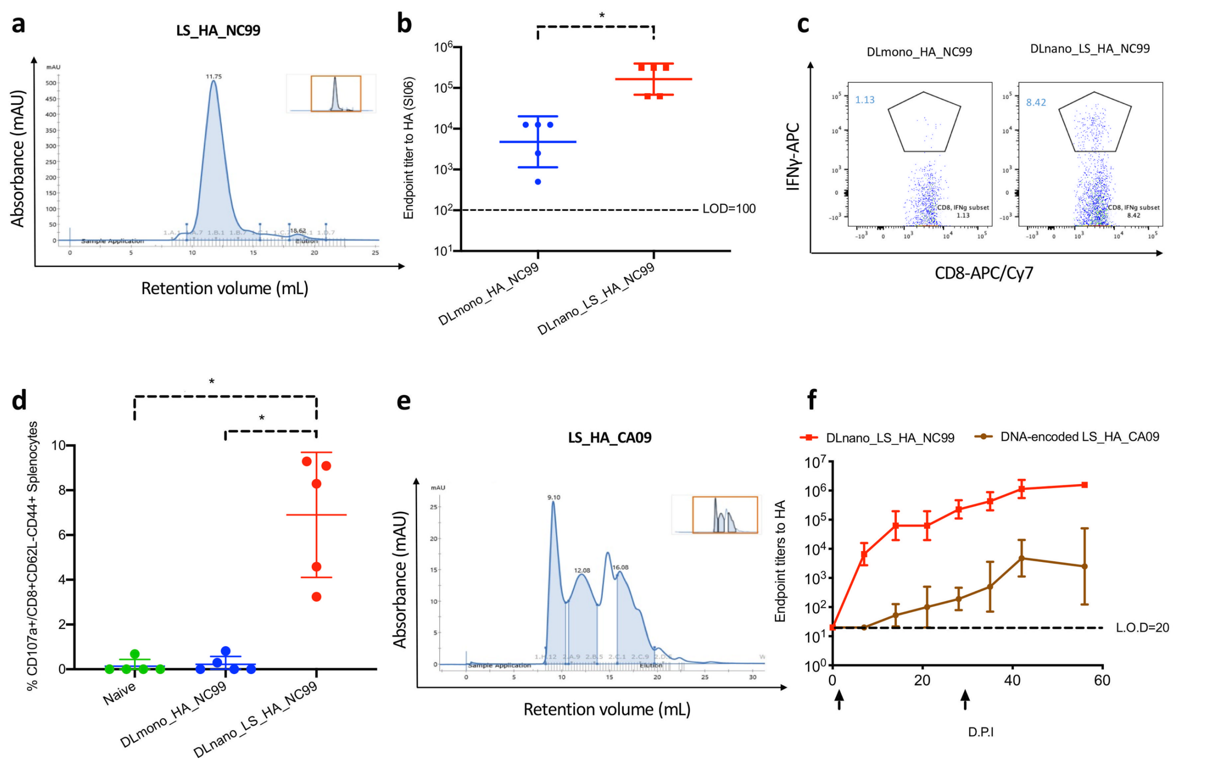

Supplemental Figure. 5. Characterization of biophysical profiles and immune responses induced by newly designed DLnano influenza hemagglutinin-based vaccines. **a.** SEC trace of recombinantly produced designed LS_HA_NC99 nanoparticles. **b.** Binding of sera from BALB/c mice immunized with 1ug DLnano_LS_HA_NC99 or DLmono_HA_NC99 at 56 d.p.i (post-dose #3) to heterologous recombinant H1 (SI06) hemagglutinin protein. **c** and **d.** CD8+ effector memory T-cell responses to NC99 HA domain in BALB/c mice immunized with two 10ug doses of DLmono_HA_NC99 or DLnano_LS_HA_NC99 in terms of IFNγ (**c.**) and CD107a (**d.**) expression. **e.** SEC trace of lectin purified recombinantly produced LS_HA_CA09. **f.** Comparison of humoral responses induced by two 10ug doses of DLnano_LS_HA_NC99, which homogeneously assembled and by DNA-encoded LS_HA_CA09, which did not homogeneously assemble *in vitro*. n=5 per group; each dot represents an animal; error bar represents standard deviation; two-tailed Mann-Whitney Rank Test used to compare groups; p-values were adjusted for multiple comparison where appropriate; *, $p<0.05$.

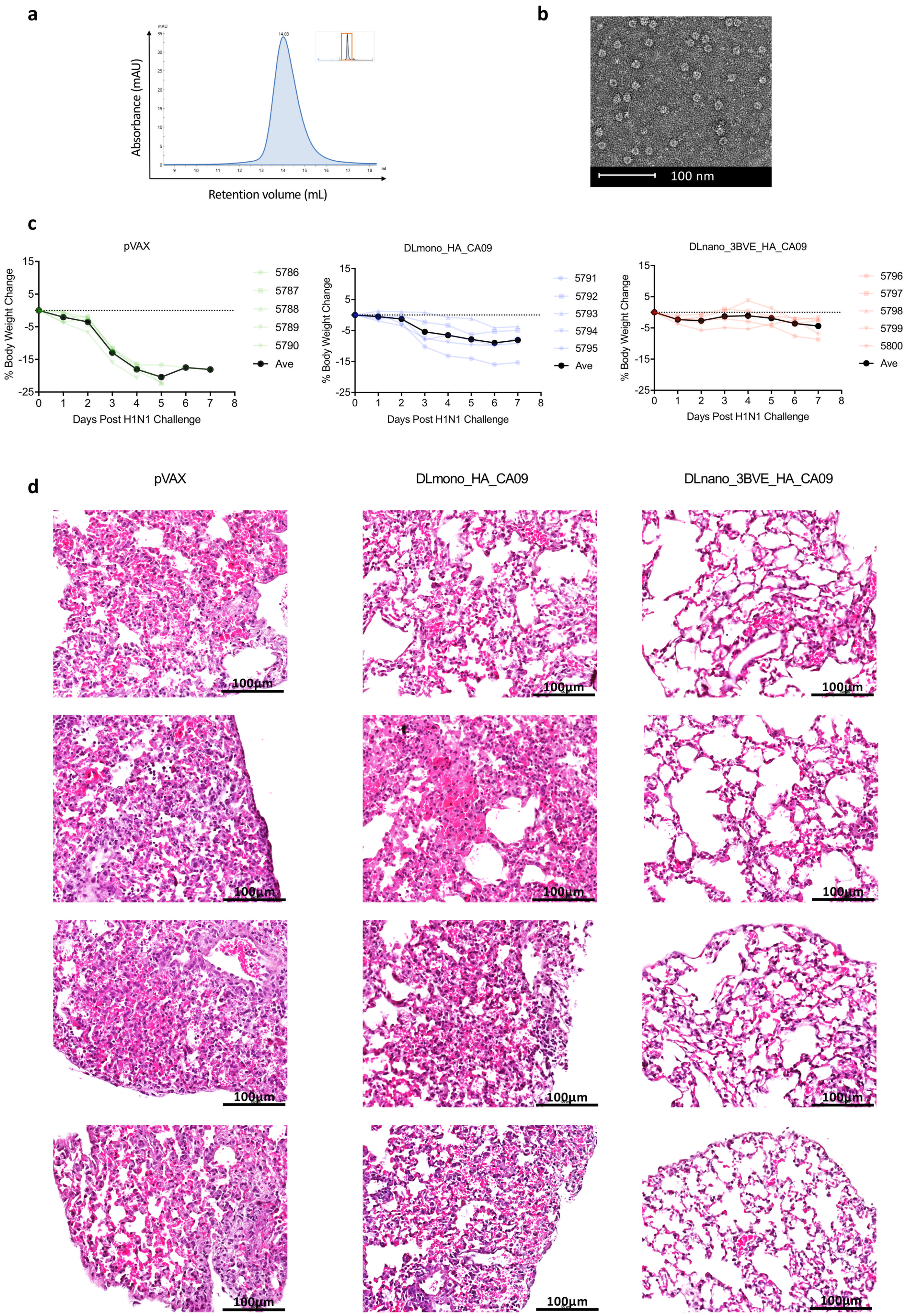

Supplemental Figure. 6. Improved protection from lethal H1/CA09 challenge in mice with DLnano_3BVE_HA_CA09 vaccination. **a.** SEC trace for lectin-purified recombinantly

produced 3BVE_HA_CA09 nanoparticles. **b.** nsEM image of SEC-purified 3BVE_HA_CA09 nanoparticles. **c.** Weight changes in mice immunized with pVAX, DLmono_HA_CA09 or DLnano_3BVE_HA_CA09 over seven-day period following 10LD_{50} H1/A/California/07/09 challenge as in **Figure. 6e**. **d.**H&E stain for lung histo-pathology in remaining 12 mice seven days post viral challenge or at the time of euthanasia as in **Figure. 6e**. 1ug DNA dose used in vivo for **c** and **d**. n=5 per group; each line represents an animal.
